# Identification of a Paracrine Signaling Mechanism Linking CD34^high^ Progenitors to the Regulation of Visceral Fat Expansion and Remodeling

**DOI:** 10.1016/j.celrep.2019.08.092

**Published:** 2019-10-08

**Authors:** Márcio Buffolo, Karla Maria Pires, Maroua Ferhat, Olesya Ilkun, Aman Makaju, Alan Achenbach, Faith Bowman, Donald L. Atkinson, William L. Holland, Ez-Zoubir Amri, Bhagirath Chaurasia, Sarah Franklin, Sihem Boudina

**Affiliations:** 1 Department of Nutrition and Integrative Physiology and Program in Molecular Medicine, University of Utah College of Health, Salt Lake City, UT 84112, USA; 2 Nora Eccles Harrison Cardiovascular Research and Training Institute, Salt Lake City, UT 84112, USA; 3 Institut de Biologie Valrose, Université Nice Sophia Antipolis, 28, avenue de Valombrose, 06107 Nice Cedex 2, France; 4 Lead Contact

## Abstract

Accumulation of visceral (VIS) is a predictor of metabolic disorders and insulin resistance. This is due in part to the limited capacity of VIS fat to buffer lipids allowing them to deposit in insulin-sensitive tissues. Mechanisms underlying selective hypertrophic growth and tissue remodeling properties of VIS fat are not well understood. We identified subsets of adipose progenitors (APs) unique to VIS fat with differential Cd34 expression and adipogenic capacity. VIS low (Cd34 low) APs are adipogenic, whereas VIS high (Cd34 high) APs are not. Furthermore, VIS high APs inhibit adipogenic differentiation of SUB and VIS low APs *in vitro* through the secretion of soluble inhibitory factor(s). The number of VIS high APs increased with adipose tissue expansion, and their abundance *in vivo* caused hypertrophic growth, fibrosis, inflammation, and metabolic dysfunction. This study unveils the presence of APs unique to VIS fat involved in the paracrine regulation of adipogenesis and tissue remodeling.

## In Brief

Buffolo et al. show that the visceral fat of mice contains subsets of Cd34 positive stromal cells with distinct adipogenic potential. Stromal cells with high Cd34 expression inhibit adipogenic conversion of cells with low Cd34 expression. When implanted *in vivo*, Cd34 high stromal cells cause visceral adipose tissue remodeling and inflammation and impair insulin sensitivity.

## Graphical Abstract

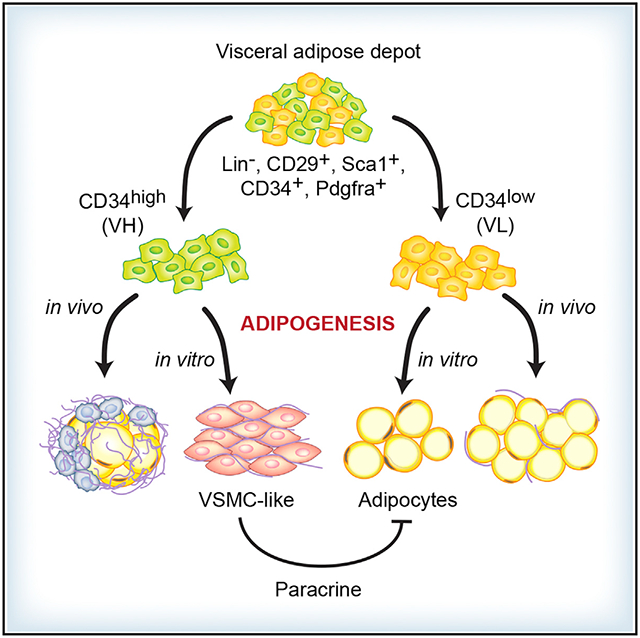

## INTRODUCTION

Obesity is a growing public health problem and is associated with cardiovascular complications (Calle et al., 1999). Visceral (VIS) obesity is a predictor of adverse metabolic and cardiovascular outcomes independently of body mass index (BMI) (Arner et al., 2013) and is associated with the development of cardio-metabolic abnormalities such as insulin resistance, glucose intolerance, and type 2 diabetes (Bastien et al., 2014). The mechanisms involved in the pathogenesis of VIS obesity are not fully understood.

White adipose tissue (WAT) expansion occurs via adipocyte hypertrophy or through *de novo* recruitment and differentiation (adipogenesis) of adipose progenitors (APs). Studies of adipogenesis in VIS and subcutaneous (SUB) fat have yielded conflicting results, with most *in vitro* studies showing increased adipogenesis in cultured SUB but not VIS APs (Grandl et al., 2016; Joe et al., 2009; Macotela et al., 2012), whereas *in vivo* studies reported enhanced adipogenesis in VIS but not in SUB fat especially in response to dietary obesity (Jeffery et al., 2015; Kim et al., 2014; Wang et al., 2013). The reasons for these discrepant findings are not currently known, but there is an agreement that, during the initial phase of WAT expansion, hypertrophic growth predominates, suggesting the existence of a mechanism that negatively regulates adipogenesis.

We and others (Hepler et al., 2018; Marcelin et al., 2017) have recently identified AP subsets unique to VIS fat of humans and mice that may be involved in the regulation of WAT expansion and remodeling. We named these CD34^+^ progenitors VIS low and VIS high APs based on their low or high expression of CD34. VIS low and VIS high APs share the same developmental origin, are distinct from SUB APs, and constitute a tissue-resident progenitor pool. However, VIS low and VIS high APs are functionally distinct. VIS low APs are committed progenitors exhibiting similar adipogenic capacity as SUB APs, whereas VIS high APs are mostly secretory and anti-adipogenic. VIS high APs secrete several soluble factors that inhibit adipogenesis. The amount of VIS high APs positively correlates with VIS adipose tissue hypertrophic expansion, and, when transplanted *in vivo*, these cells exacerbate diet-induced obesity and insulin resistance. This study provides evidence for a tissue-resident APs unique to VIS fat of mice that are involved in the regulation of adipose tissue expansion and remodeling in response to caloric excess.

## RESULTS

### Murine VIS Fat in Mice Contains Two AP Subsets with Distinct Adipogenic Capacity

**Figure 1.**
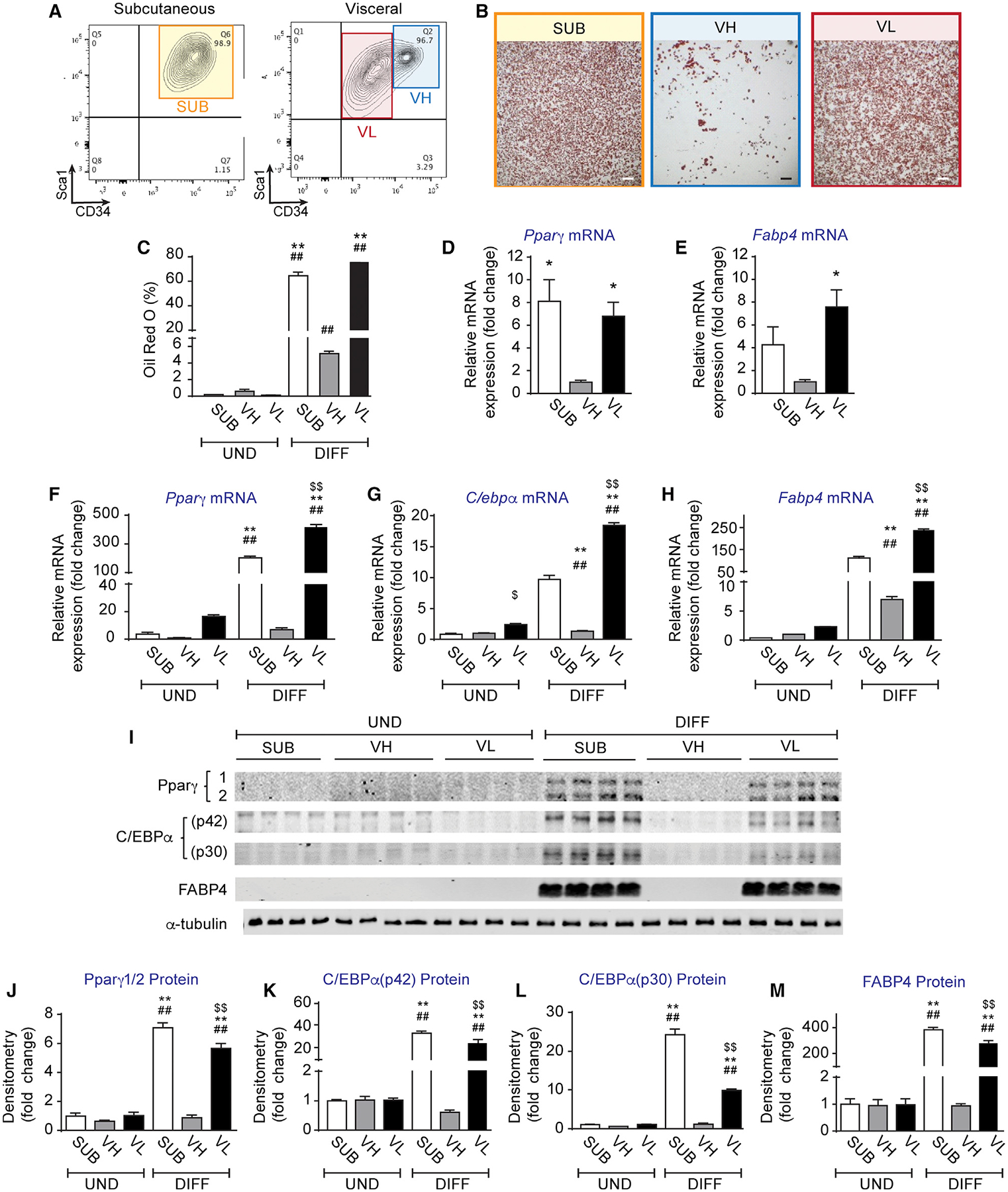
Distinct Adipogenic Potential among Visceral Adipose Tissue Progenitors (A) FACS plots of Lin^−^, CD29^+^ Sca1^+^, and CD34^+^ adipose progenitors (APs) in subcutaneous (inguinal) and visceral (epididymal) adipose tissue of C57BL/6 control mice. (B) Representative images of oil red O staining of differentiated subcutaneous (SUB), visceral low (VL), and visceral high (VH) APs. (C) Oil red O quantification expressed as percentage of staining. (D and E) Relative mRNA expression of (D) *Pparγ* and (E) *Fabp4* in sorted not cultured VH, SUB, and VL AP subsets, respectively, expressed as fold change from VH. (F–H) Relative mRNA expression of (F) *Pparγ*, (G) *C/ebpα*, and (H) *Fabp4* in undifferentiated (UND) and 6-days-differentiated (DIFF) VH, SUB, and VL APs, respectively, expressed as fold change from VH. (I) Representative Western blots of Pparγ, C/ebpα, Fabp4, and α-Tubulin expression in UND and 6-days-DIFF VH, SUB, and VL APs. (J–M) Densitometry of (J) Pparγ, (K) C/ebpα (p42), (L) C/ebpα (p30), and (M) Fabp4 protein expression normalized by α-Tubulin and expressed as fold change from VH. Values are mean ± SEM (*p < 0.05; **p < 0.005 versus VH under the same condition; ##p < 0.005 versus UND within the same progenitor subset; $ $p < 0.005 versus SUB under the same condition (n = 3–4 independent experiments, each including 10–15 mice each). Scale bar in (B) is 400 μm.

Using a previously established fluorescence-activated cell sorting (FACS) strategy (Figure S1) to isolate WAT APs from C57BL/6 (B6) mice (Rodeheffer et al., 2008), we identified two major cell populations in VIS fat (eWAT) of B6 mice distinguished by their differential expression of CD34 (Figure 1A). We separately sorted Lin^−^, CD29^+^, and Sca1^+^ APs that displayed either low (VIS low) or high (VIS high) expression of CD34. As compared to Lin^−^, CD29^+^, Sca1^+^, and CD34^+^ APs from SUB, VIS low APs showed comparable adipogenic potential (Figures 1B and 1C). In contrast, VIS high APs were refractory to adipogenesis (Figures 1B and 1C). Consistent with lipid accumulation, SUB and VIS low APs expressed 8- and 7-fold more *Pparγ* mRNA, respectively, as compared to VIS high APs (Figure 1D). Similarly, these cells had far greater expression of the Pparγ target *Fabp4*, which was enhanced by 4- and 8-fold in SUB and VIS low APs, respectively, as compared to VIS high APs (Figure 1E). To verify whether these differences in adipogenic regulators persisted in cultured and differentiated AP subsets, we sorted SUB, VIS low, and VIS APs and differentiated them in the presence of a differentiation media (DM) containing dexamethasone, insulin, and IBMX for 3 days followed by growth media for 3 more days. We confirmed that SUB and VIS low, but not VIS high APs, displayed an enhanced response to this differentiation protocol, as they significantly increased mRNA (Figures 1F–1H) and protein (Figures 1I–1M) expression of Pparγ, C/ebpα, and Fabp4. These results are consistent with a recent report showing that a subset of murine VIS APs with differential expression of CD9 exhibited distinct adipogenic capacity (Marcelin et al., 2017). Indeed, when we examined CD9 expression in our APs, we found that most of VIS high cells were CD9^+^, whereas most of VIS low cells were CD9^−^ both in B6 and CH3 strains (Figures S1P–S1R). Similarly, it was recently suggested that Ly6c can distinguish adipogenic versus non-adipogenic VIS APs (Hepler et al., 2018). However, we showed that Ly6c may not be a good marker for adipogenic VIS low or SUB APs as most of these cells are Ly6c^+^ (Figures S1K–S1M). Finally, VIS low and VIS high APs are also present in other VIS depots (Figures S2A and S2B), and their adipogenic property is conserved across several mouse strains (Figures S2C–S2H).

### VIS Low and VIS High APs Share the Same Developmental Origin and Are Tissue-Resident Progenitors

SUB and VIS WAT are known to exhibit differential expression of developmental genes, suggesting divergent origins (Gesta et al., 2006). Indeed, some VIS APs were recently shown to originate from the mesothelium in the lateral plate mesoderm, an origin that was distinct from SUB APs (Chau et al., 2014). VIS low and VIS high APs may have originated from the mesothelium, during development as evidenced by the high mRNA expression of the mesothelial markers Wilms tumor 1 (*Wt1*), mesothelin (*Msln*), and uroplakin 3B (*Upk3b*), respectively, when compared to SUB APs (Figures S2I–S2K). Consistent with previous reports (de Jong et al., 2015; Macotela et al., 2012), VIS APs also expressed higher levels of the mesoderm gene *Tcf21* (Figure S2L), thus making it a good marker for VIS progenitors. It is worth noting that, while we did not see any difference in *Pdgfrα* mRNA expression between VIS APs and SUB APs (Figure S2M), VIS APs expressed higher mRNA levels of the mural cell marker *Pdgfrβ*, suggesting a mesoderm-mural cell origin for VIS, but not for SUB progenitors (Figure S2O). Finally, as CD24 marked Pdgfrα^+^ APs that were less committed (Berry and Rodeheffer, 2013), we investigated its expression in VIS high APs. Surprisingly, VIS low and VIS high APs were largely CD24^−^, though both contained a small fraction of CD24^+^ cells (Figures S1I–S1J). Furthermore, *Cd24* mRNA expression, although similar in VIS low and VIS high APs, it was 10-fold lower in SUB APs (Figure S2P).

Prior studies demonstrated that *de novo* production of bona fide adipocytes from bone marrow (BM)-derived progenitors could occur both in humans and mice (Rydén et al., 2015). We thus tested the possibility that VIS low and VIS high APs may have derived from circulating progenitors by performing a BM transplantation from B6 UBC-GFP transgenic donor mice into irradiated recipient B6 mice. After confirming the transplantation of blood cells (Figure S1S), we subjected the recipient mice to either a normal chow diet (NCD) or a high-fat diet (HFD) for 7 weeks before sorting VIS high and VIS low APs. Independent of the diet, we found that >90% of VIS low and VIS high APs were GFP^−^ (Figures S1T–S1W), indicating that these cells were tissue-resident APs.

### The Adipogenic Potential of VIS High APs Can Be Increased by Rosiglitazone or BMP4

Inhibition of adipogenesis in VIS APs that were not separated based on CD34 expression was improved by treatment with rosiglitazone or BMP4 *in vitro* (Joe et al., 2009; Macotela et al., 2012). Therefore, we sought to test whether these molecules could rescue the differentiation defect in VIS high APs. VIS low and VIS high APs underwent differentiation in the presence or absence of rosiglitazone or BMP4. These agents significantly increased adipogenesis in VIS high APs as measured by *4,4-difluoro-4-bora-3a,4a-diaza-s-indacene* (BODIPY) staining and Pparγ and Fabp4 protein expression (Figures S3A–S3C). However, adipogenesis was far lower when compared to VIS low APs (Figures S3D–S3M). These results suggest that VIS high APs have the potential to differentiate into adipocytes although less than VIS low APs.

We next examined whether signaling pathways known to suppress or promote adipogenesis were altered in VIS high APs. Thus, we investigated the anti-adipogenic Wnt/β-catenin signaling (Rosen and MacDougald, 2006). However, we found no difference in β-catenin expression between VIS high and VIS low APs (data not shown). Finally, to exclude the possibility that VS high APs were unable to respond to DM, we measured phosphorylation of insulin receptor β (IRβ), Akt, and Creb after 24 h of differentiation. We found a significant 31% reduction in IRβ tyrosine phosphorylation in VIS high APs compared to VIS low APs (Figures S3N and S3O). However, this reduction did not translate into diminished downstream signaling to Akt (Figures S3N and S3P–S3Q). Interestingly, we observed a 2-fold increase in Creb phosphorylation in undifferentiated VIS low APs versus undifferentiated VIS high APs, but both cells similarly reduced Creb phosphorylation upon differentiation (Figures S3N and S3R). Taken together, these data suggest that the block in adipogenesis in VIS high APs is unlikely to be caused by enhanced Wnt signaling or impaired IR downstream signaling.

### VIS High APs Inhibit Adipogenesis of VIS Low and SUB APs *In Vitro* via the Secretion of Soluble Factor(s)

**Figure 2.**
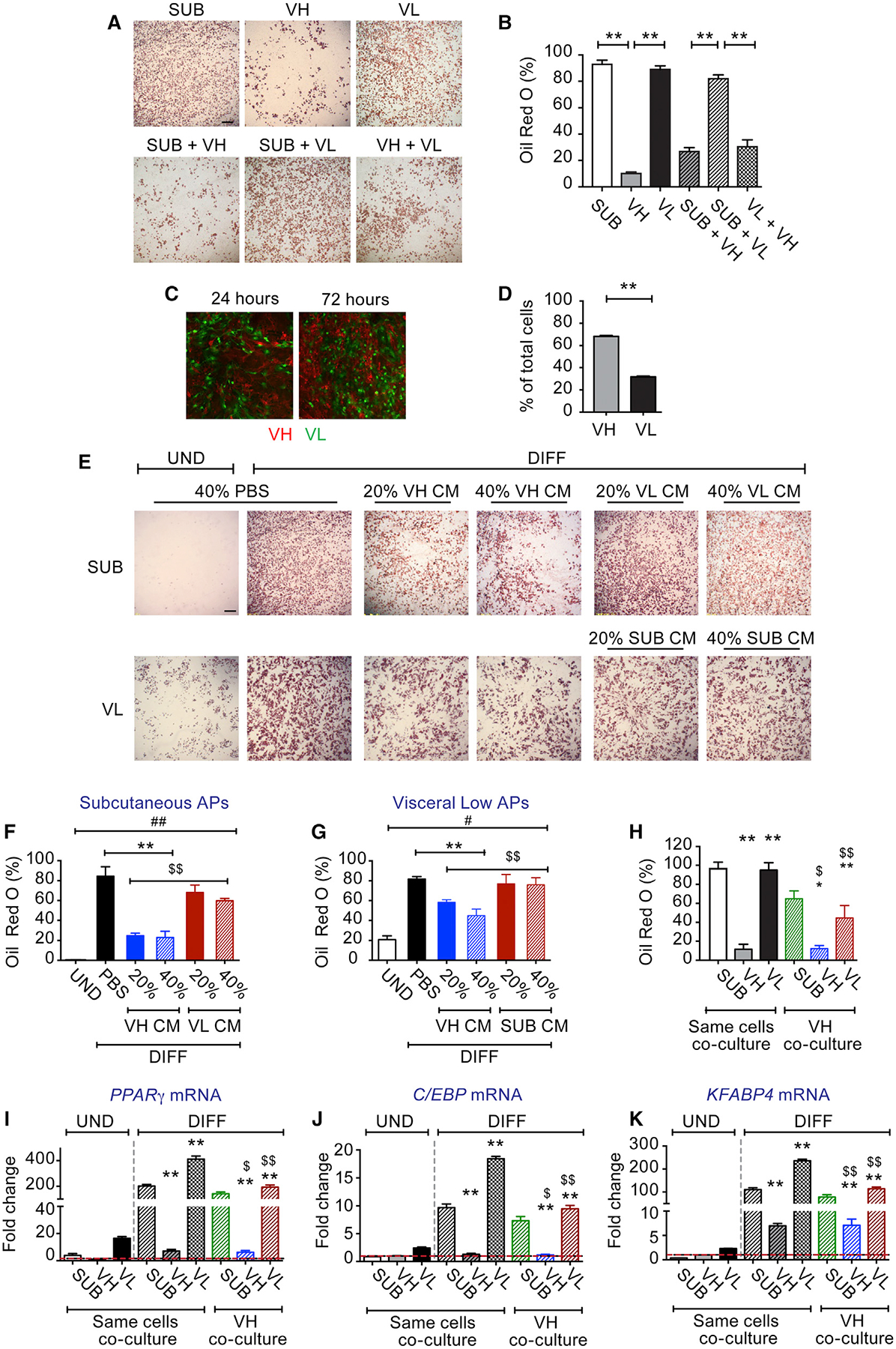
Visceral High APs Inhibit Differentiation of Visceral Low and SUB APs (A) Representative images of oil red O staining of AP co-cultures DIFF for 6 days. VH, SUB, and VL APs were cultured alone or with equal amounts of VH APs. (B) Oil red O staining quantification expressed as percentage of staining. (C) Representative images of co-cultures of UND VL (obtained from UBC-GFP transgenic mice) and VH (obtained from mT/mG transgenic mice) APs for 24 and 72 h, respectively. (D) Flow cytometry quantification of green (VL) and red cells (VH) expressed as percentage of total cells 72 h post-co-culture. (E) Representative images of oil red O staining of SUB and VL AP cultures treated during the first 3 days of differentiation with PBS or with different concentrations of conditioned media (CM) from 3-days-DIFF VH cultures. (F and G) The corresponding quantification of oil red O staining expressed as percentage of staining in (F) SUB and (G) VL APs treated with PBS or with CM from VH, respectively. (H) Quantification of oil red O staining expressed as percentage of staining in transwell co-cultures. VH, SUB, and VL APs were co-cultured on a transwell system either with the same AP subtype or with VH APs on top. Cells were DIFF for 6 days, and the bottom cultures were stained with oil red O. (I–K) Relative mRNA expression of (I) *Pparγ*, (J) *C/ebp*α, and (K) *Fabp4*, respectively, in UND and DIFF transwell co-cultures. Values are mean ± SEM (*p < 0.05; **p < 0.005 versus UND VH; $p < 0.05; $ $p < 0.005 versus same cell co-cultures under the same condition; n = 3–4 independent experiments, each including 10–15 mice each). Scale bar in (A) and (E) is 400 μm and in (C) is 200 μm.

Previous studies showed that, when VIS low and high APs were not separated, the cells had poor differentiation (Joe et al., 2009; Macotela et al., 2012), suggesting that VIS high APs may inhibit the adipogenic capacity of VIS low APs. To directly test that, we co-cultured equal amounts of VIS high APs with either VIS low or SUB APs before subjecting them to DM. As shown in Figures 2A and 2B, VIS low or SUB APs cultured alone differentiated very well. In contrast, co-culturing them with VIS high APs significantly suppressed adipogenesis (Figures 2A and 2B). To investigate the mechanisms underlying this adipogenic block, we first examined whether VIS high APs had higher proliferation rates and thus might simply outnumbered VIS low or SUB APs following a prolonged culture. To test this, we isolated VIS low APs from B6 UBC-GFP transgenic mice and co-cultured them with an equal number of VIS high APs isolated from transgenic B6 mice harboring membrane-targeted tdTomato transgene (mT/mG mice). The use of GFP-labeled VIS low APs and tdTomato-labeled VIS high APs allowed us to quantify their proportions using flow cytometry. Consistent with our hypothesis, we observed a significant 2-fold increase in the number of VIS high APs compared to VIS low APs at confluence (day 3 of culture) (Figures 2C and 2D), revealing the higher proliferative potential of VIS high APs. Nonetheless, this disparity could not explain the 3-fold decrease in oil red O staining observed in the co-culture experiments (Figure 2B). Thus, we sought to determine whether VIS high APs secrete factor(s) that inhibit adipogenic differentiation. We collected conditioned media (CM) from cultured VIS high, VIS low, or SUB APs. We then treated separate cultures of VIS low, VIS high, or SUB APs with 20% or 40% of CM or similar volume of PBS. CM from VIS high markedly impaired differentiation of VIS low and SUB APs (Figures 2E–2G), suggesting that VIS high APs secrete factor(s) that inhibit adipogenic differentiation. Because co-cultures can induce cell-to-cell contact inhibition of proliferation and therefore influence differentiation (Grandl et al., 2016), we also evaluated this hypothesis using a transwell system that allowed cells to be co-cultured without contact, sharing soluble factors through a permeable membrane. As shown in Figures 2H–2K, co-culturing VIS low or SUB APs with VIS high APs in the upper chamber of the transwell system resulted in the suppression of adipogenesis. These data confirmed that VIS high APs not only proliferate faster but negatively influence the adipogenic capacity of both VIS low and SUB APs through the secretion of soluble factor(s).

**Figure 3.**
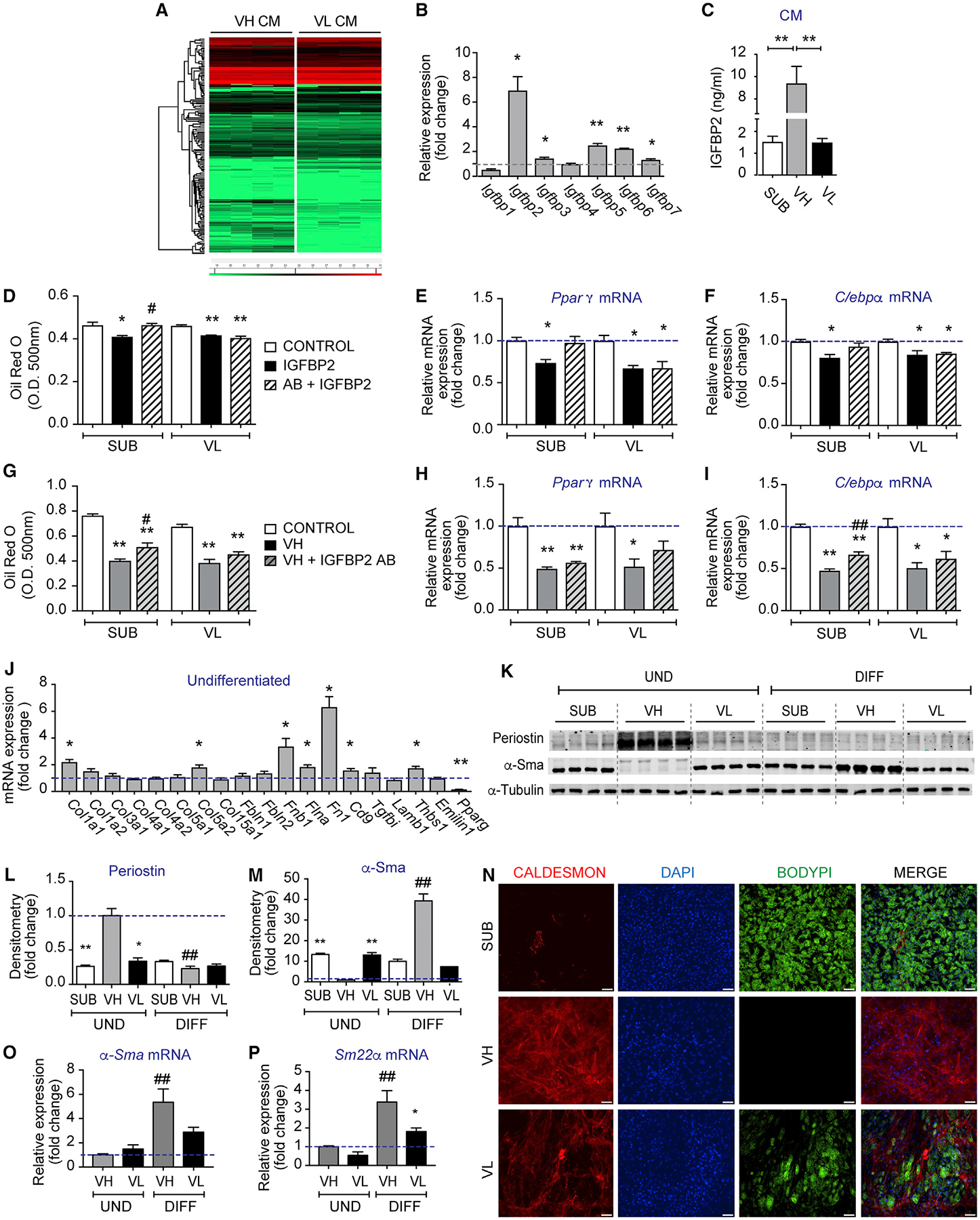
Visceral High APs Secrete Adipogenic Inhibitors and Acquired a Smooth Muscle Fate upon Adipogenic Differentiation (A) Hierarchical clustering of differentially abundant proteins in CM from 3-days-DIFF VH and VL APs, respectively. (B) Relative mRNA expression of Igfbp1–7 in VIS low and VIS high APs at day 2 post-differentiation. Levels are expressed as fold change relative to VIS low APs. (C) Igfbp2 levels in CM of SUB, VIS low, and VIS high cultures at day 6 post-differentiation. (D–F) Oil red O quantification (D) and relative mRNA expression of *Pparγ* (E) and *C/ebpα* (F) in SUB and VIS low AP cultures DIFF in the presence of PBS control, 25 ng/mL mouse recombinant Igfbp2 alone or combined with 30 μg/mL Igfbp2 neutralizing antibody during the first 3 days of differentiation. (G–I) Oil red O quantification (G) and relative mRNA expression of *Pparγ* (H) and *C/ebpα* (I) in SUB and VIS low AP cultures DIFF in the presence of 20% of PBS or VIS high CM from cells treated or not with 30 μg/mL Igfbp2 neutralizing antibody during differentiation. (J) Relative mRNA expression of genes encoding extracellular matrix (ECM) components and fibroblast markers in 6 days UND VIS low and VIS high APs expressed as fold change from VIS low. (K) Representative Western blots of periostin, α-Sma, and α-Tubulin in UND and 6-days-DIFF SUB, VH, and VL APs, respectively. (L and M) The corresponding densitometry of (L) periostin and (M) α-Sma expression normalized by α-Tubulin and expressed as fold change from UND VH. (N) Representative images of immunocytochemistry in 6-days-DIFF SUB, VH, and VL APs stained for caldesmon, 4′,6-diamidino-2-phenylindole (DAPI), and BODIPY. (O and P) Relative mRNA expression of (O) *α-Sma* and (P) *Sm22*a in UND and 6 days DIFF VH and VL APs expressed as fold change from UND VH. Values are mean ± SEM (*p < 0.05; **p < 0.005 versus VH under the same condition; #p < 0.05; ##p < 0.005 versus UND within the same progenitors subset; $p <0.05; $ $p < 0.005 versus SUB under the same condition (n = 3–4 independent experiments, each including 10–15 mice). Scale bar size is 50 μm.

We next sought to identify abundant proteins in CM from 3-days-differentiated VIS high and VIS low APs using proteomics (PXD014672). As depicted in Figures 3A and S4A, we found that VIS high APs secreted more factors than VIS low APs. Among the most significantly (≥R2-fold) abundant proteins in CM of VIS low APs were angiotensinogen (Agt), lipocalin 2 (Lcn2), and retinol-binding protein 4 (Rbp4), which we did an ELISA and confirmed its presence in CM of VIS low cells (Figures S4A and S4B). The presence of these proteins in CM of VIS low APs is consistent with their conversion to adipocytes. In contrast, VIS high APs secreted mostly extracellular matrix (ECM) and cell adhesion molecules and some cytokines (Figures 3A and S4A). Some of the secreted proteins identified in CM of VIS high APs were previously shown to inhibit adipogenesis, including fibronectin, se-crosteonectin, and tissue inhibitor of metalloproteinase 1 (Timp1) (Meissburger et al., 2011; Nie and Sage, 2009; Spiegelman and Ginty, 1983). However, none of these factors inhibited the differentiation of VIS low or SUB APs in our hands (data not shown). Furthermore, previous studies showed that aortic carboxypeptidase-like protein (Aclp) inhibited adipogenic differentiation of 3T3-F442A preadipocytes, 10T1/2 mouse cells, and human preadipocytes (Abderrahim-Ferkoune et al., 2004; Jager et al., 2018). Thus, we overexpressed Aclp in Expi293 cells, confirmed its secretion (Figure S4D), and treated SUB and VIS low cultures with 10% of CM obtained from Aclp-overexpressing cells or from empty vector-expressing cells prior to or post-differentiation. Treatment with CM containing Aclp had no effect on adipogenesis (Figures S4E and S4F).

Other abundant proteins in CM of VIS high APs are insulin-like growth factor proteins (Igfbps). Because of the known involvement of Igfbp2 in obesity and fat accumulation and due to the 7-fold increase in its mRNA expression in VIS high cells (Figure 3B), we sought to determine its role in adipogenesis. We first measured Igfbp2 protein content post-differentiation and found it highly abundant in CM of VIS high APs (Figure 3C). We then treated SUB and VIS low cultures with recombinant mouse Igfbp2 during the first 3 days of differentiation and assessed adipogenesis. Treatment with Igfbp2 modestly reduced lipid accumulation and *Pparγ* and *C/ebpα* mRNA expression in SUB and VIS low cultures, and this effect was reversed by the addition of Igfbp2 neutralizing antibody only in SUB APs (Figures 3D–3F). Next, we treated SUB and VIS low AP cultures with CM collected from VIS high APs differentiated in the presence or absence of the Igfbp2 neutralizing antibody. As shown in Figures 3G–3I, neutralizing Igfbp2 action had minimal effect on lipid accumulation and adipogenic gene expression in SUB and VIS low AP cultures (Figures 3G and 3H). Taken together, these data suggest that, while VIS high cells secrete high amounts of Igfbp2, this factor alone is not sufficient to inhibit adipogenesis. Interestingly and despite the slight increase in Igfbp6 mRNA in VIS high APs, the secretion of this protein was rather enhanced in CM of SUB followed by VIS high and VIS low, respectively (Figure S4I). The increase in Igfbp2 secretion by VIS high APs and the abundance of these cells in obesity prompted us to examine its level in the serum of lean and obese (ob/ob) mice. Igfbp2 levels were significantly reduced in the serum of obese mice as opposed to Rbp4 levels, which were increased (Figures S4J and S4K). Last, as Igfbps are known to regulate the level of insulin-like growth factors (Igfs), we measured Igf-1 levels in CM of SUB, VIS low, and VIS high APs at day 6 post-differentiation. Consistent with the secretion of Igfbp2, VIS high APs secreted higher amount of Igf-1 as opposed to the other APs (Figure S4L). Together, these results demonstrate that VIS high APs secrete high levels of Igfbp2 and Igf-1 but Igfbp2 alone had a modest inhibitory effect on adipogenesis.

### VIS High APs Have Characteristics of Fibroblast Progenitors Capable of Acquiring a Vascular Smooth Muscle-like Phenotype upon Adipogenic Differentiation *In Vitro*

The secretome profile of VIS high APs suggested that these cells are involved in ECM remodeling. As fibroblasts and myofibroblasts are known to secrete several of the above-mentioned molecules, we measured mRNA expression of several collagens and ECM molecules and found that undifferentiated VIS high APs had higher expression of *Col1a1*, *Col5a2*, *Fnb1*, *Flna*, and *Fn1* as compared to undifferentiated VIS low APs (Figure 3J). Some of these genes maintained higher expression after 6 days of differentiation (Figure S4C). These results suggest that VIS high APs are fibroblastic in nature. To explore whether the non-adipogenic VIS high APs differentiated into vascular smooth muscle cells (VSMCs) *in vitro*, we examined VSMC markers. Undifferentiated VIS high APs expressed 3-fold higher levels of the fibroblast and myofibroblast marker periostin (Snider et al., 2009) when compared to SUB and VIS low APs, respectively (Figures 3K and 3L). However, periostin content was abolished after differentiation in all AP subtypes (Figures 3K and 3L). Contrary to periostin, the VSMCs marker α-Sma (Wang et al., 2006), which was absent in undifferentiated VIS high APs, was significantly induced in these cells after differentiation (Figures 3K and 3M). These results were further confirmed by increased *α-Sma* mRNA expression in differentiated VIS high but not VIS low APs (Figure 3O). The absence of periostin and the induction of α-Sma seems contradictory as the expression of these proteins coincide in myofibroblasts in the heart (Kanisicak et al., 2016). This result led us to speculate that VIS high APs may have converted to VSMCs instead, since these cells also express α-Sma (Wang et al., 2006). We, therefore, examined additional markers for VSMCs such as Sm22α and found that VIS high APs had high mRNA expression of this marker upon differentiation (Figure 3P). Finally, the higher expression of caldesmon protein examined by immunocytochemistry or by Western blot further confirmed that VIS APs acquired a VSMC-like phenotype (Figures 3N, S4G, and S4H). How might VIS high APs sense the changes in ECM to undergo VSMC fate switch is currently unknown but may involve integrin and focal adhesion signaling as previously demonstrated for other mesenchymal stem cells and pre-adipocytes. These results are highly significant as researchers routinely mix VIS low and VIS high APs in culture *in vitro*, resulting in a mixture of cells with different fates.

### Altered VIS Low and VIS High APs Proportions during WAT Expansion

**Figure 4.**
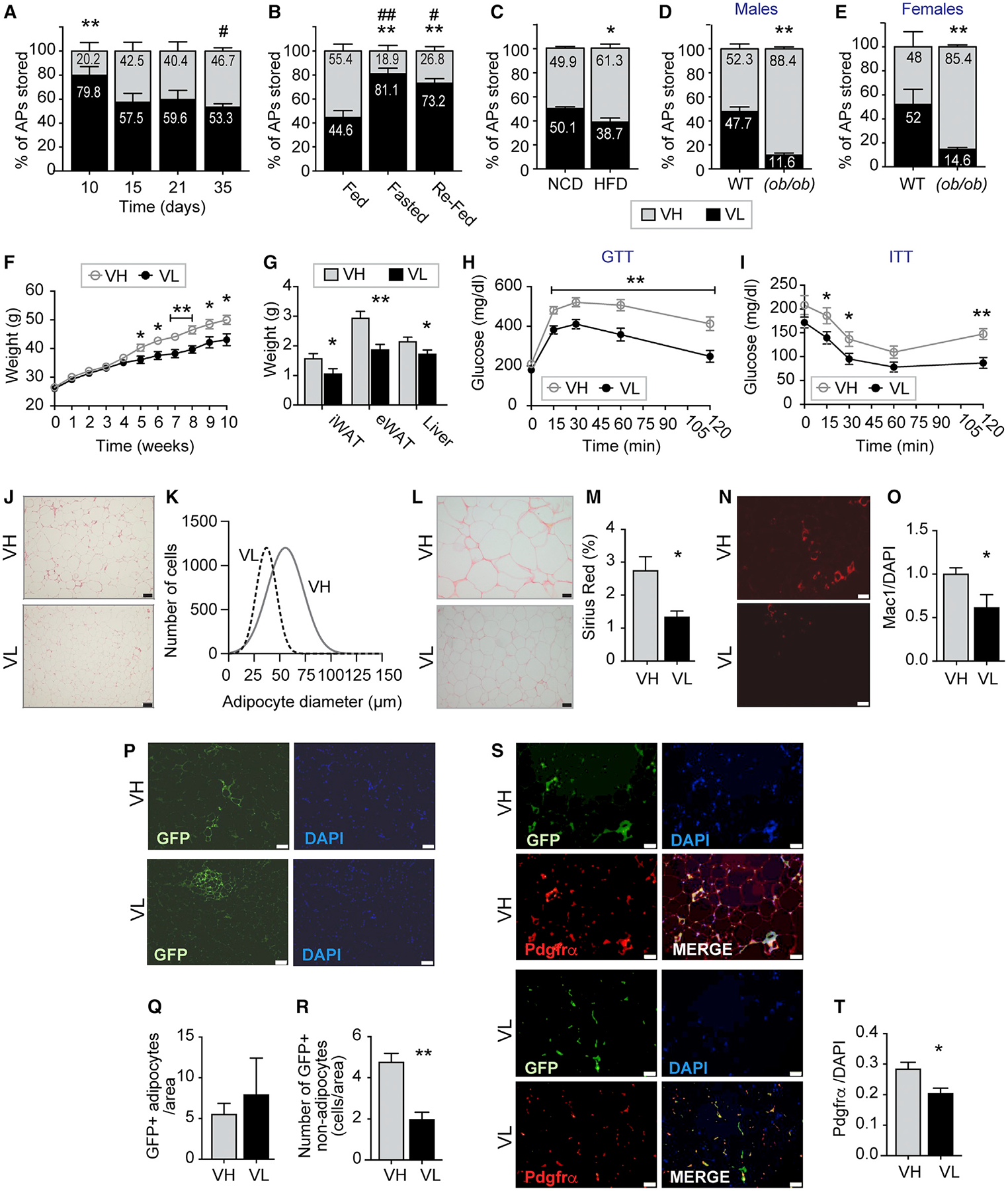
Visceral High AP Proportions Correlate Positively with Visceral Adipose Tissue Healthy and Unhealthy Expansion (A) Visceral high and low AP proportions in C57BL/6 male mice at post-natal day 10, 15, 21, and 35, respectively. (B) Visceral high and low AP proportions in C57BL/6 male mice fed, fasted, or fasted and then refed. (C) Visceral high and low AP proportions in C57BL/6 male mice fed normal chow diet (NCD) or high-fat diet (HFD) for 7 weeks. (D and E) Visceral high and low AP proportions in male (D) and female (E) (*ob/ob*) and wild-type mice, respectively. (F–T) Visceral low and high APs were isolated from eWAT of UBC-GFP transgenic male mice and transplanted into C57BL/6 recipient mice. Mice were fed HFD for 10 weeks before being sacrificed. (F) Body weights in transplanted mice. (G) Inguinal (iWAT), epididymal (eWAT), brown adipose tissue (BAT), and liver weights normalized by body weights in C57BL/6 recipient mice. (H) Glucose tolerance test (GTT) of VIS low and VIS transplanted mice. (I) Insulin tolerance test (ITT) of VIS low and VIS transplanted mice. (J) Representative images of eWAT sections of VIS low and VIS transplanted mice stained with H&E. (K) Adipocyte diameter in eWAT of VIS low and VIS transplanted mice. (L and M) Representative images of eWAT of VIS low and VIS transplanted mice stained with Sirius Red (L) and the corresponding quantification (M) expressed as percentage. (N and O) Representative images of eWAT of VIS low and VIS transplanted mice stained with Mac1 antibody (N) and the corresponding quantification (O) normalized by DAPI. (P–R) Representative images of eWAT of VIS low and VIS high transplanted mice stained with GFP antibody (P) and quantification of GFP-labeled adipocytes (Q) and non-adipocytes (R) normalized by DAPI. (S and T) Representative images of eWAT of VIS low and VIS transplanted mice stained with Pdgfrα antibody (S) and the corresponding quantification (T) normalized by DAPI. Values are mean ± SEM (*p < 0.05; **p < 0.005 versus VH under the same condition; #p < 0.05; ##p < 0.005 versus 10 days, fed or NCD within the same progenitors subset; n = 5–12 mice per group). Scale bar size is 50 μm.

We demonstrated that VIS high APs negatively regulate adipogenesis *in vitro*; however, it is still unknown whether these cells influence adipogenesis and WAT expansion *in vivo*. Thus, we first examined the proportions of VIS AP subsets during physiologic (postnatal and fasting and refeeding) and pathologic (diet-induced and genetic obesity) fat expansion. Since most of VIS fat expansion occurs postnatally (Chau et al., 2014), we isolated stromal vascular fractions form eWAT of B6 mice at post-natal day 10, 15, 21, and 35 (P10, P15, P21, and P35), sorted the APs, and determined the percentage of VIS low and VIS high APs by flow cytometry. At P10, 80% of VIS progenitors were VIS low (Figure 4A). In contrast, equal proportions of VIS low and VIS high APs were visible at P15 and remained that way until adulthood (Figure 4A). These results argue against VIS high APs being the precursors of VIS low APs. Next, we investigated whether the reduction in adipose tissue mass by fasting affected the number of these cells. We subjected B6 mice to 72 h fasting followed by refeeding for 5 days (Figure S5A). This fasting protocol reduced body and epididymal WAT (eWAT) weights and adipocyte size, which were then partially restored by refeeding (Figures S5B–S5D). Fasting resulted in a significant reduction in VIS high APs, whereas refeeding partially increased it (Figure 4B). The altered distribution of VIS low and VIS high APs during fasting-refeeding could result from changes in their turnover. Thus, we measured bromodeoxyuridine (BrdU) incorporation in VIS low and VIS high APs and observed a trend toward more BrdU^+^ incorporation in both VIS low and VIS high APs during refeeding (Figures S5T and S5U).

Dietary obesity was previously shown to affect the proliferative rate of APs in VIS fat depot but not in SUB fat depot of mice (Jeffery et al., 2015; Joe et al., 2009; Lee et al., 2012; Marcelin et al., 2017). Therefore, we examined whether the proportions VIS APs would be affected by both dietary and genetic (leptin-deficient ob/ob) obesity. Seven weeks of HFD resulted in a 50% increase in weight gain and eWAT mass (Figures S5E and S5F), which was associated with an increase in adipocyte hypertrophy and a decrease in hyperplasia (Figures S5G–S5I). HFD shifted AP proportions toward more VIS high APs (Figure 4C). Furthermore, this shift was more dramatic and occurred in both male and female (ob/ob) mice (Figures 4D and 4E), suggesting that obesity favors the proliferation of VIS high APs or loss of VIS low APs. However, we found that VIS high APs did not incorporate more BrdU when compared to VIS low APs (Figures S5T and S5U). Our results on proliferation are not consistent with previous reports (Jeffery et al., 2015). This could be due to the fact that proliferation occurred earlier during the course of the diet and we only measured it after 7 weeks.

### VIS High APs Exacerbate Diet-Induced Weight Gain and Insulin Resistance and Promote Adipose Tissue Remodeling *In Vivo*

While our *in vitro* studies suggest that VIS high APs are not adipogenic, the contribution of these cells to fat formation *in vivo* is unknown. Thus, we transplanted VIS low or VIS high APs (10^5^ cells of each) isolated from eWAT of B6 UBC-GFP transgenic mice into eWAT of B6 recipient mice using Matrigel. The recipient mice for both VIS low or VIS high APs were allowed to recover from surgery before starting HFD feeding regiment for 10 weeks. Body weights, eWAT, and inguinal WAT (iWAT) weights were significantly increased in mice transplanted with VIS high APs when compared to mice transplanted with VIS low APs (Figures 4F and 4G). Mice receiving VIS high APs were more glucose and insulin intolerant when compared to mice transplanted with VIS low APs (Figures 4H and 4I). Adipocyte diameter was increased in eWAT of mice receiving VIS high APs compared to the ones receiving VIS low APs (Figures 4J and 4K). We also observed more collagen deposition in eWAT of mice receiving VIS high APs as measured by Sirius Red staining (Figures 4L–4M). Furthermore, eWAT of mice receiving VIS high APs contained more macrophages as measured by immunofluorescence using the macrophage marker mac-1 (Figures 4N and 4O). To gain insights into the weight gain caused by VIS high transplantation, we monitored metabolic activity, movement, and food intake in NCD and HFD mice over a 72 h period at 6 weeks of age (a time when body weights were not different).

Consistent with weight gain, mice transplanted with VIS high and maintained on HFD exhibited lower metabolic activity as evidenced by the significant decrease in whole-body oxygen consumption and CO_2_ production (Figures S5N and S5O). While food intake was not significantly different between the HFD groups, activity was slightly lower in mice transplanted with VIS high and maintained on HFD (Figures S5R and S5S). To determine the fate of VIS low and VIS high APs *in vivo*, we examined GFP labeling of adipocytes and non-adipocytes in eWAT of recipient mice. An equivalent number of GFP-labeled adipocytes were detected in eWAT of mice transplanted with VIS low or with VIS high APs, respectively (Figures 4P–4R). However, mice receiving VIS low APs exhibited clusters of GFP-positive adipocytes, whereas dispersed GFP-labeled adipocytes were detected in mice receiving VIS low APs. Contrary to the equivalent number of GFP-positive adipocytes, we observed a higher number of GFP-labeled stromal cells (non-adipocytes) in eWAT of mice transplanted with VIS high APs (Figures 4P–4R). To gain insights into the localization of VIS low and VIS high APs, we stained eWAT sections from transplanted mice with Pdgfrα. As shown in Figures 4S and 4T, eWAT from mice transplanted with VIS high APs had higher number of Pdgfrα^+^ stromal cells, most of which were GFP^+^. Altogether, these results demonstrated that, under HFD, VIS high APs were capable of forming adipocytes but most importantly give rise to stromal cells. The nature of these stromal cells originating from VIS high APs is currently unknown and is the subject of future investigations.

## DISCUSSION

Although CD34^+^ progenitors have been extensively characterized as adipocytes progenitors (Berry and Rodeheffer, 2013; Díaz-Flores et al., 2014; Zwierzina et al., 2015), our study and those of others (Hepler et al., 2018; Marcelin et al., 2017) have now demonstrated that, within the pool of CD34^+^ APs in VIS of mice, there exists a subset of CD34^high^ cells with anti-adipogenic, pro-fibrotic, and pro-inflammatory properties. Our study further showed that CD34^high^ (VIS high) APs secrete high levels of Igfbp2, but this protein had minimal effect on adipogenesis *in vitro*. *In vivo*, VIS APs are capable of forming dispersed adipocytes but most importantly promote tissue fibrosis and inflammation during HFD. Because CD34 expression was shown to decrease during differentiation (Suga et al., 2009), we reasoned that VIS low APs may have originated from a stem-like VIS high progenitors. However, our *in vitro* and *in vivo* data do not support this idea. Indeed, VIS high APs cannot spontaneously differentiate into adipocytes despite long culture times, which should reduce CD34 expression. In addition, the proportions of VIS low APs exceed those of VIS high APs during post-natal growth of eWAT (Figure 4A). Finally, the expression of Cd24, previously shown to mark uncommitted fat progenitors, was similar in VIS low and VIS high APs.

Similar cells were recently found by others in VIS fat of humans and mice and were isolated based on CD9 or Ly6c expression, respectively (Hepler et al., 2018; Marcelin et al., 2017). Based on the cell-surface markers we used, we believe that the cells we identified are similar to those reported by Marcelin et al. (2017). However, there are some differences between the APs described here and those identified by Hepler et al. (2018). Although, they both express Pdgfrβ^+^, VIS low and VIS high APs cannot be distinguished based on Ly6c expression. Furthermore, the use of SUB APs in our study revealed that these cells are Ly6c^high^, but they still maintain a good adipogenic potential, suggesting that this marker may not be suitable for distinguishing adipogenic from non-adipogenic APs. The higher expression of mesothelial markers in VIS low and VIS high APs also suggests that these cells may have multiple developmental origins. Indeed, single-cell sequencing of Pdgfrβ^+^ stromal cells revealed four distinct clusters of cells, with one expressing mesothelial cell markers (Hepler et al., 2018). In addition, a previous study showed that a subset of VIS but not SUB adipocytes originated from Wt1^+^ stromal cells (Chau et al., 2014). Further studies are needed to better define the developmental origin of VIS low and VIS high APs.

The rate by which *de novo* formation of fat cells is tightly regulated, and there is an agreement that hypertrophic growth as opposed to hyperplastic growth of a fat depot directly correlate with metabolic health (Denis and Obin, 2013; Klöting et al., 2010). Indeed, hypertrophic growth of WAT is associated with cell death, inflammation, and fibrosis, all of which may influence the lipid buffering capacity of this tissue. It has been observed both in humans and in mice that hypertrophic growth, inflammation, and fibrosis are predominant in VIS fat depots when compared to SUB fat depots, but the underlying mechanisms are not well understood. Therefore, we reasoned that VIS fat may contain an intrinsic paracrine mechanism to regulate fat expansion. Our results now unveiled a mechanism by which VIS fat expansion can be regulated at the cellular level. We found that VIS high APs inhibit adipogenic differentiation of both SUB and VIS low APs *in vitro*.

Igf-1 has been shown to play an important role in adipocyte proliferation and differentiation, and its bioavailability is modulated by six homologous Igf-binding proteins (Igfbps) (Smith et al., 1988). Among all other Igfbps examined in this study, *Igfbp2* mRNA expression was an ~7-fold increase in VIS high APs. Despite the abundance of Igfbp2 in CM of VIS high APs, the effect of this protein on adipogenesis was modest. One explanation for these results could be that Igfbp2 alone is not sufficient to inhibit adipogenesis. Moreover, the inhibitory effect on adipogenesis by Igfbp2 is likely independent of downstream Igf-1 signaling since no difference in Akt signaling was observed between VIS low and VIS high APs (Figures S4Q). Consistent with our results, other studies showed that Igfbp2 inhibits adipogenesis of preadipocytes in an Igf-1-independent manner (Britton et al., 2013; Wheatcroft et al., 2007). Although, we showed that Igfbp2 is highly secreted by VIS high APs, we still don’t know the significance of this secretion to WAT expansion and remodeling. Prior studies involving transgenic mice overexpressing human IGFBP2 showed that the mice resisted diet-induced weight gain and insulin resistance (Wheatcroft et al., 2007). Similarly, several reports and our own data showed that serum IGFBP2 are reduced in obese humans and mice (Frystyk et al., 1999; Li and Picard, 2010). These findings are counterintuitive, but it is possible that tissue secretion as opposed to circulating levels may modulate adipogenesis; however, this needs to be further confirmed. Furthermore, the inhibition of adiogenesis by Igfbp2 was modest as opposed to the robust inhibition observed in culture. Thus, it is possible that Igfbp2 may not be the sole adipogenic inhibitor secreted by VIS high APs or that this protein cooperates with other factors to fully inhibit differentiation. Indeed, it was shown that Igfbp2 cooperates with vimentin to mediate Igf-1 receptor action in VSMCs (Shen et al., 2015). More studies are needed to fully characterize the mechanism underlying adipogenesis inhibition by VIS high APs.

In conclusion, this study provides important insights into the function of AP subsets that we and others have discovered (Hepler et al., 2018; Marcelin et al., 2017). Although heterogeneity in VIS progenitors has long been evoked (Rosen and MacDougald, 2006), a functional role for different AP populations in VIS fat was lacking. Here, we showed that VIS high APs have the capacity to modulate adipogenesis of other progenitors. It is tempting to speculate that VIS high APs may constitute a niche that control VIS low APs stemness and proliferation by blocking their differentiation. This, in turn, may explain why VIS but not SUB fat maintains a dynamic AP population that can be mobilized during the expansion of the depot. Our study also reveals a role for VIS high APs in the induction of tissue fibrosis and inflammation following HFD.

One key observation from the current study is the capacity of VIS high APs to differentiate into VSMCs. This is consistent with the mass spectrometry data showing that VIS high APs secrete Aclp, a protein known to increase during VSMCs differentiation (Meissburger et al., 2011). Moreover, overexpression of Aclp in 3T3-F442A preadipocytes was shown to induce VSMC-like differentiation (Abderrahim-Ferkoune et al., 2004). The mechanisms involved in this lineage commitment change are currently unknown, but prior studies have shown that Aclp-induced α-Sma expression in lung fibroblasts was mediated by Tgfβ/smad signaling. From these results, however, it is unclear whether this fate switch occurs *in vivo* but nevertheless suggests that VIS high APs are capable of multi-lineages differentiation. The development of lineage tracing strategies to specifically track VIS low and VIS high APs *in vivo* is the next step toward understanding their function *in vivo*.

### Study Limitation

Although several laboratories including ours have identified subsets of CD34^+^ stromal cells in VIS adipose tissue of humans and mice with distinct adipogenic potential, the mechanisms underlying this difference in adipogenesis are not fully understood. We acknowledge that the present study has several limitations including the use of 2D cultures and the *in vitro* setting to assess adipogenesis. In addition, the use of Matrigel in the transplantation experiment may have affected the properties of the transplanted cells. Finally, the mechanisms underlying weight gain and metabolic dysfunction in mice transplanted with VIS high are currently unknown. Future studies will focus on developing tools to allow lineage tracing of VIS low and VIS high APs and to examine their tissue location, adipogenic potential, and fibrogenic and immunogenic behavior *in vivo*.

## STAR★METHODS

### LEAD CONTACT AND MATERIALS AVAILABILITY

Further information and requests for resources and reagents should be directed to and will be fulfilled by the lead contact, Sihem Boudina (Sihem.boudina@u2m2.utah.edu). This study did not generate new unique reagents.

### EXPERIMENTAL MODEL AND SUBJECT DETAILS

Mice were maintained under standard laboratory conditions at a temperature of 22 ± 2°C, relative humidity of 50 ± 5% and photo-period of 12h (12hdark and 12h-light cycle). All animal experiments were performed according to procedures approved by University of Utah Institutional Animal Care and Use Committee and adhered to NIH standards. Experimental mice were individually caged and only male mice were used for the experiments except for Figure 4E that included female mice. The ages of all mice used in the studies ranged between 9–10 weeks except for Figure 4, where pups were used at ages 10–35 days. All animals were used in scientific experiments for the first time. This includes no previous exposures to pharmacological substances. High fat diets were provided to the indicated mice to induce obesity. Health status was normal for all animals.

A total of 440 adult male mice were used. Strain details and number of animals in each group are as follows: 300 C57BL/6J; 20 UBC-GFP Tg mice; 10 mT/mG mice; 50 FVB/NJ mice; 50 CH3 mice and 10 (*ob/ob*) mice.

### METHOD DETAILS

#### Animals and Diets

For high-fat diet feeding (HFD) studies, 6–9-week old male C57BL/6J male mice consumed normal chow diet (NCD) (Research Diets Inc., New Brunswick, NJ) containing (kilocalories) 10% fat, 70% carbohydrate and 20% protein or HFD containing (kilocalories) 60% fat, 20% carbohydrate, and 20% proteins for 7 or 10 weeks. For fasting and re-feeding experiments, C57BL/6J male mice were fasted for 72 h or fasted for 72 h and then re-fed for 5 days. Leptin-deficient (*ob/ob*) male and female mice were used at 9 weeks of age.

#### Adipose Progenitors (APs) Isolation and Differentiation

Stromal vascular cells (SVCs) were isolated from inguinal, epididymal, or retroperitoneal white adipose tissue (WAT) by collagenase digestion as previously described (Wheatcroft et al., 2007). The SVC was treated with red blood cells lysis buffer, suspended in HBSS and labeled with antibodies against cell surface markers previously identified to mark APs (Rodeheffer et al., 2008). APs were separated by fluorescence-activated cell sorting (FACS) under sterile conditions and cultured in growth media (GM) consisting of DMEMF-12 supplemented with 10% FBS, 100 U/ml penicillin, 100 μg/ml streptomycin and 10 ng/ml bFGF. For BMP and rosiglitazone treatment, APs were treated with 3.3 nmol/L BMP-4 or 1 μmol/L rosiglitazone during the first 3 days of differentiation.

For conditioned media (CM) treatments, APs were exposed to various concentrations of CM or equal volume of PBS during the first 3 days of differentiation. Sorted APs were seeded at 50,000/cm^2^ in GM and cultured for 3 days without changing the media. At 100% confluence (3–4 days), the cells were exposed to adipogenic differentiation media (DM) containing 1 μg/ml insulin, 0.25 mg/ml dexamethasone and 0.5 mmol/L isobutylmethylxanthine (IBMX) in DMEM-F12 with 10% FBS and 1% penicillin/streptomycin. After 3 days in DM, APs were switched to GM and cultured for 3 more days before staining neutral lipids with Oil Red O.

#### Oil Red O and BODIPY staining

Cells were fixed in 4% paraformaldehyde for 15 min at room temperature, then rinsed three times with PBS. Oil Red O (Oil Red O Kit, Abcam) staining was done according to the manufacturer instructions. Cells were stained with BODIBY (10 μg/ml) for 1 h at room temperature. Wells were washed three times with PBS and then mounted with ProLong Gold Antifade Mountant with DAPI.

#### Bone Marrow Transplantation

Tibias and femurs of 6 weeks old UBC-GFP transgenic male mice were used. Both ends of all bones were trimmed to allow insertion of a 26-gauge needle to flush the bone marrow with DMEM medium. This tissue was harvested by alternately flushing with medium and scratching the bone marrow cavity with the end of the needle. Clumps of bone marrow cells were gently dissociated to form a single-cell suspension. Single-cell suspensions were gently centrifuged at 1500 rpm for 10 min at 4°C to obtain a cell pellet. One day prior to transplantation, 6-weeks old male C57BL/6J recipient mice were lethally irradiated with 2 doses of 500 cGy split approximately 4–6 h apart as previously described (Yau et al., 2015). Irradiated animals were allowed to rest in cages with unlimited food and water for two hours prior to bone marrow injection. Mice were immobilized using an animal holder and tail veins located. Recipient mice received (7.5 × 10^5^ cells/ml) injected intravenously. Mice were then fed a normal chow diet (NCD) or high fat diet (45% HFD) for 7 weeks. Mice were euthanized, the fat was processed and the APs were sorted as previously described.

#### Histology and Morphometry

Epididymal (eWAT) depot was fixed in 4% paraformaldehyde for 48 h, embedded in paraffin, cut into 5-μm sections, and stained with Hematoxylin and Eosin (H&E). The adipocyte diameter was determined as previously described (Wheatcroft et al., 2007). The total number of adipocytes was measured using the CellSens Software. The images were acquired with an XM10 Olympus fluorescent camera.

#### Immunocytochemistry and Immunohistochemistry

Cells were fixed in 4% paraformaldehyde for 15 min at room temperature, then rinsed three times in PBS. Permeabilization was done using a 0.5% Triton X-100 in PBS solution at room temperature for 5 min. Cells were blocked with 1% BSA, 22.52 mg/mL glycine in PBST (PBS+ 0.1% Tween 20) for 30 min. Cells were incubated overnight with caldesmon (1:100) diluted in 1% BSA in PBST at 4°C. After, antibody solution was aspirated and cells were washed three times in PBS (5 min each wash). Cells were incubated with the secondary antibody (Alexa Fluor 594, 1:500 in 1% BSA PBST) for 1 h at room temperature. Epididymal white adipose tissue (eWAT) from transplanted mice was fixed in 4% paraformaldehyde, embedded in paraffin and cut into 5-μm sections. Slides were allowed to dry for 48 h before antigen retrieval with Tris-EDTA at 100°C for 30 min. Slides were then incubated with blocking solution (1% bovine serum albumin, 1% goat serum, 22.52 mg/ml glycine in PBS with 1% Tween-20. Slides were then incubated with either anti-GFP, anti-Mac1 or anti-Pdgfra antibody at room temperature for 1 h then at 4°C overnight. Wells or slides were washed three times in PBS and then mounted with ProLong Gold Antifade Mountant with DAPI. Images were acquired using an inverted IX-71 Olympus epi-fluorescence microscope equipped with a digital XM-10 camera and Cell Sense software package (Olympus, Valley, PA).

#### Quantification of Proliferating Cells by BrdU

A single Brdu injection (2 mg/mL, i.p.) was administered 24 h after the start of the fasting protocol and mice were euthanized after 72 h later. For the fasted/refed group, Brdu was injected on day 2 after the refeeding protocol was initiated. Mice were euthanized after 5 days of refeeding. For HFD group, BrdU injection was given 6 weeks after the diet protocol was initiated and mice were sacrificed at week 7 of the diet. APs were sorted and fixed in 2% paraformaldehyde for 15 min and BrdU staining was performed according to the manufacturer protocol. BrdU positive or apoptotic cells were determined for each subpopulation of APs by flow cytometry.

#### Adipose Progenitors Transplantation

UBC-GFP Tg mice at 8 week of age were used to sort VIS low and VIS high APs from eWAT as described above. VIS high or VIS low APs (5 × 10^4^ cells for each subset), were mixed with BD Matrigel Basement Membrane Matrix and injected in eWAT fat pad of 8 week-old C57BL/6J recipient male mice. After a recovery period of 1 week, recipient mice received a normal chow diet (NCD) or a high fat diet (60% HFD) for 8–10 weeks.

#### Glucose and Insulin Tolerance Tests

Mice were fasted four hours (for ITT) or six hours (for GTT) respectively starting in the morning (8–9am). Baseline (fasting) blood glucose was measured before insulin or glucose administration from venous blood via a small tail tip cut. Insulin (1U/kg body weight) (ITT) or Glucose (2 g/kg body weight) (GTT) (2 g/kg body weight) was injected i.p. Blood glucose values were obtained at 15 30, 60, 90 and 120 min from the initial tail cut.

#### Indirect Calorimetry

Mice were housed in a four-chamber Oxymax Comprehensive Lab Animal Monitoring System (CLAMS) (Columbus Instruments, Columbus, OH) for 72 h at 23°C. The O2 and CO2 content of the exhaust air from each chamber were compared with the ambient air O2 and CO2 content. Food consumption was monitored using an electronic scale, water by an electronic sipper tube, and movement by XY/Z laser beam interruption. The respiratory exchange ratio (RER) was calculated as VCO2/VO2.

#### Western Blot Analysis

Cells were homogenized in cell lysis buffer (30 mM HEPES pH 7.4, 1% NP-40, 1 mM EDTA, 1 mM dithiothreitol, 10% glycerol, and protease inhibitor cocktail (Sigma, St. Louis, Mo, USA)). The protein concentration was measured using a Micro BCA reagent (Pierce, Rockford, IL, USA). The protein extracts were resolved by SDS-PAGE and electro-transferred onto an Immobilon PVDF membrane (Millipore Corp., Bedford, MA, USA). Alexa Fluor anti-rabbit 680 (Invitrogen, Carlsbad, CA, USA) and anti-mouse 800 (VWR International, West Chester, PA, USA) were used as the secondary antibodies. Fluorescence was quantified using the LICOR Odyssey imager (LI-COR, Lincoln, NE, USA).

#### Gene Expression Analysis

Total RNA was isolated with TRIzol (Life Technologies, Grand Island, NY, USA) according to the manufacturer’s instructions. For the quantitative PCR, the final reaction volume was 10 μl and included specific primers, 10 ng of cDNA and the SYBR green master mix (Life Technologies, Grand Island, NY, USA). The real-time PCR assays were run on an ABI Prism 7900HT real-time PCR machine (Applied Biosystems, USA). Normalization was performed using ribosomal protein L13 (*Rpl13*) RNA. Quantification was performed using the comparative ΔCt method.

#### LC-MS/MS Proteomics

In-solution digestion of conditioned media (CM) proteins was carried out using the Filter-Aided Sample preparation method (Frystyk et al., 1999). Briefly samples were mixed with 20 μl of SDT buffer and heated to 80°C for 10 min. Samples were then centrifuged at 16000 g and the supernatant was loaded into 10KD Vivacon 500 filter units, centrifuged at 13000 g for 20 min, and then the concentrated protein was washed three times with 200 μL UA buffer via centrifugation. The concentrate was then mixed with 100 μL 50 mM iodoacetamide in UA buffer and incubated in darkness at room temperature for 20 min, followed by centrifugation for 15 min. The concentrate was subsequently washed twice with 100 μL UA buffer followed by two washes with 100 μL 50 mM ammonium bicarbonate. Sequencing grade trypsin was added at a 1:40 ratio and incubated overnight at 37°C. The peptides were then collected by centrifugation at 13000 g for 15 min, the filters were washed with 50 μl mass spectrometry grade water and the collected peptides were acidified with 1% formic acid. SDT buffer: 4% SDS, 100mM DTT, 100mM Tris/HCl pH 7.6. UA buffer: 8M Urea, 0.1M Tris/HCl pH 8.5

Tryptic peptides were analyzed by nanoflow LC-MS/MS on a Thermo Orbitrap Velos Pro coupled with a Thermo EASY-nLC 1000 using a reversed-phase column (75μm inner diameter 15cm, Reprosil C-18 AQUA 3μm particle size; New Objective) and a flow rate of 400 nL/min. For peptide separation, a multi-step gradient was utilized from 98% Buffer A (0.1% formic acid, 5% DMSO) and 2% Buffer B (0.1% formic acid, 5% DMSO in acetonitrile) to 65% Buffer A and 35% Buffer B over 145 min. The spectra were acquired using Nth order double-play, data-dependent acquisition mode for the top 20 most abundant ions in the parent spectra for fragmentation. MS1 scans were acquired in the Orbitrap mass analyzer at a resolution of 60000. MS1 ions were fragmented by CID fragmentation with an activation time of 10 ms and a normalized collision energy of 35. Dynamic Exclusion was enabled for 60 s intervals to avoid multiple fragmentations of parent ions.

The raw data files were analyzed using MaxQuant (Li and Picard, 2010) and searched against the Uniprot mouse database. The database search was carried out using the in-built Andromeda search engine and label-free quantitation was carried out using the MaxLFQ module (Shen et al., 2015). A mass tolerance of 20 ppm for precursor ions and 0.5 Da for fragmented ions was used during the search. The peptides were searched for the static modification of carbamidomethylation on cysteine and the variable modification of methionine oxidation and protein N-terminal acetylation. Peptides were filtered using a FDR < 1 and protein quantitation was carried out using the LFQ intensities generated for each protein.

### QUANTIFICATION AND STATISTICAL ANALYSIS

Data are presented as the mean ± SEM; p < 0.05 were considered significant. Student’s t test was used to compare two independent groups. One-way or two-way ANOVA with Tukey post hoc test were used for multiple group analysis. N values are indicated in the figure legends and refer to biologically independent replicates. Statistical and bioinformatics analysis of the mass spectrometry data was carried out using Perseus (Tyanova et al., 2016).

## Supplementary Material

1

## Figures and Tables

**Table:** KEY RESOURCES TABLE

REAGENT or RESOURCE	SOURCE	IDENTIFIER
Antibodies		
Rabbit anti-PPARγ	Cell Signaling Technology	Cat#2435; RRID: AB_2166051
Rabbit anti-C/EBPα	Cell Signaling Technology	Cat#8178; RRID: AB_11178517
Rabbit anti-FABP4	Cell Signaling Technology	Cat#3544; RRID: AB_2278257
Mouse anti-AKT(pan	Cell Signaling Technology	Cat#2920; RRID: AB_1147620
Rabbit anti-p(S473) AKT	Cell Signaling Technology	Cat#4060; RRID: AB_2315049
Rabbit anti-p(T308)AKT	Cell Signaling Technology	Cat#9275; RRID: AB_329828
Mouse anti-CREB	Cell Signaling Technology	Cat#9104; RRID: AB_490881
Rabbit anti-p(S133)CREB	Cell Signaling Technology	Cat#9198; RRID: AB_2561044
Rabbit anti-IRβ	Cell Signaling Technology	Cat#3025; RRID: AB_2280448
Rabbit anti-p(Y1131)IGF1β/ p(Y1146)IRβ	Cell Signaling Technology	Cat#3021; RRID: AB_331578
Rabbit anti-c-Myc	Cell Signaling Technology	Cat#9402; RRID: AB_2151827
Mouse anti-ACLP/AEBP1	Santa Cruz Biotechnology	Cat#sc-271374; RRID: AB_10608716
Mouse anti-α-Tubulin	Sigma	Cat#T6199; RRID: AB_477583
Mouse anti-Periostin	Sigma	Cat#SAB4200197; RRID: AB_10669144
Rabbit anti-Igfbp2	Fisher Scientific	Cat#MAB797; RRID: AB_2264599
Rabbit anti- αSMA	Abcam	Cat#ab5694; RRID: AB_2223021
Rabbit anti-Caldesmon	Abcam	Cat#ab32330; RRID: AB_725810
Rabbit anti-PDGFRα	Abcam	Cat#ab124392; RRID: AB_10978090
Biotin anti-mouse TER-119	BioLegend	Cat#116204; RRID: AB_313705
Biotin anti-mouse CD45	BioLegend	Cat#103104; RRID: AB_312969
Biotin anti-mouse CD31	BioLegend	Cat#102404; RRID: AB_312899
PE/Cy7 anti-mouse/rat CD29	BioLegend	Cat#102222; RRID: AB_528790
PE anti-mouse CD140a (PDGFR-α)	BioLegend	Cat#135906; RRID: AB_1953269
PerCP/Cy5.5 anti-mouse CD24	BioLegend	Cat#101824; RRID: AB_1595491
Antibodies (continued)		
Alexa Fluor647 mouse anti-CD9	BioLegend	Cat#124810; RRID: AB_2076037
Rilliant Violet 421mouse anti-Ly-6C	BioLegend	Cat#128032; RRID: AB_2562178
CD44 rat anti-mouse CD44, V500	BD Biosciences	Cat#560780; RRID: AB_1937316
Ly-6A/E(Sca-1) rat anti-mouse, V500	BD Biosciences	Cat#561228; RRID: AB_10584334
PE rat anti-mouse CD34	BD Biosciences	Cat#551387; RRID: AB_394176
GFP Tag Polyclonal antibody	Thermo Fisher Scientific	Cat#A-21312; RRID: AB_221478
Chemicals, Peptides, and Recombinant Proteins		
Matrix Matrigel High Prot	VWR/Genemate	CAS: 80094–330
BODIPY 650/665-X NHS Ester (Succinimidyl Ester)	Invitrogen/Thermo Fisher	CAS: D10001
Recombinant Mouse IGFBP-2 (carrier- free)	Biolegend	CAS: 750304
FITC Streptavidin	Biolegend	CAS: 405202
SYTOX BlUE dead cell stain	Invitrogen	CAS: S34857
DAPI Nuclear Counterstain	Thermo Fisher	CAS: 62248
BMP4 Recombinant Human Protein	Invitrogen	CAS: PHC9534
Rosiglitazone	Sigma	CAS: R2408
Critical Commercial Assays		
IGFBP-2 ELISA Kit, Mouse	Invitrogen	CAS: EMIGFBP2
IGFBP-6 ELISA Kit, Mouse	Invitrogen	CAS: EMIGFBP6
Mouse RBP4 Quantikine ELISA Kit	Fisher Scientific	CAS: MRBP40
Mouse IGF-I ELISA Kit	Sigma	CAS: RAB0229
FITC BRDU FLOW KIT	BD biosciences	559619
Experimental Models: Organisms/Strains Deposited Data		
C57BL/6J	The Jackson Laboratory	#000664
C57BL/6-Tg(UBC-GFP)	The Jackson Laboratory	#004353
B6.129(Cg)-Gt(ROSA)26SOR (mT/mG)	The Jackson Laboratory	#007676
FVB/NJ	The Jackson Laboratory	#001800
C3H/HeJ	The Jackson Laboratory	#000659
B6.Cg-*Lep* ^ob^/J (ob/ob)	The Jackson Laboratory	#000632
Oligonucleotides		
Primers – *Pparγ:* F- *GCCTATGAGCACTTCACAAGAAAT*	This Paper	N/A
Primers – *Pparγ:* R- *GGAATGCGAGTGGTCTTCCA*	This Paper	N/A
Oligonucleotides (Continued)		
Primers – *C/ebpα:* F- *GCAAAGCCAAGAAGTCGGTG*	This Paper	N/A
Primers – *C/ebpα:* R- *TCACTGGTCAACTCCAGCAC*	This Paper	N/A
Primers – *Fabp4:* F- *TGAAATCACCGCAGACGACA*	This Paper	N/A
Primers – *Fabp4:* R- *ACACATTCCACCACCAGCTT*	This Paper	N/A
Primers – *Col1a1*: F- *CGCTGGTCAAGATGGTC*	This Paper	N/A
Primers – *Col1a1*: R- *CTCCAGCCTTTCCAGGTTCT*	This Paper	N/A
Primers – *Col1a2:* F- *AGCCCTGGTTCTCGAGGT*	This Paper	N/A
Primers – *Col1a2:* R- *CCGGTTGAACCACGATTG*	This Paper	N/A
Primers – *Col3a1*: F- *CTCCTGGTGAGCGAGGAC*	This Paper	N/A
Primers – *Col3a1*: R- *GACCAGGTTGCCCATCACT*	This Paper	N/A
Primers – *Col4a1*: F- *TGGCACAAAAGGGACGAG*	This Paper	N/A
Primers – *Col4a1*: R- *GGCCAGGAATACCAGGAAGT*	This Paper	N/A
Primers – *Col4a2:* F- *CCCGGATCTGTACAAGGGTG*	This Paper	N/A
Primers – *Col4a2:* R- *CGCCTTTTGAGATTACGCCG*	This Paper	N/A
Primers – *Col5a1*: F- *CAAGAAGTCTGAGGGAGCCA*	This Paper	N/A
Primers – *Col5a1*: R- *ACGCTTGAATTCACTGAACCAG*	This Paper	N/A
Oligonucleotides (Continued)		
Primers – *Fbln1:* F- *GACACCTTCCGCCAAGAGAA*	This Paper	N/A
Primers – *Fbln1*: R- *ATGATCTCCTCAGGACGGGT*	This Paper	N/A
*Oligonucleotides (continued): See* Table S1.		
Deposited Data		
Mass spectrometry data	ProteomeXchange Consortium via the PRIDE [1] partner repository	Accession # PXD014672
Software and Algorithms		
Graphpad Prism 7	Graphpad	http://www.graphpad.com/scientific-software/prism/
CellSens	Olympus	https://www.olympus-lifescience.com/en/software/cellsens/
GeneTools	Syngene Bioimaging	http://www.syngene.com/genetools/
Graphpad Prism 7	Graphpad	http://www.graphpad.com/scientific-%20software/prism/

## Data Availability

Proteomics data reported in this paper are deposited in ProteomeXchange data base with the accession number PXD014672.

## Highlights

- Visceral fat contains subsets of stromal cells with distinct adipogenic potential
- Cd34^high^ stromal cells inhibit adipogenesis through the secretion of soluble factors
- Cd34^high^ stromal cells cause tissue remodeling and metabolic dysfunction *in vivo*
