# Supplementary material for: Identification of a Paracrine Signaling Mechanism Linking CD34high Progenitors to the Regulation of Visceral Fat Expansion and Remodeling
Source: Cell Rep. Author manuscript; Available in PMC 2024 Mar 19. (PMC10950319; doi:10.1016/j.celrep.2019.08.092)
Supplement: 1 [file NIHMS1872850-supplement-1.pdf]

Cell Reports, Volume *29*

Supplemental Information

Identification of a Paracrine Signaling Mechanism Linking CD34^{high} Progenitors to the Regulation of Visceral Fat Expansion and Remodeling

Márcio Buffolo, Karla Maria Pires, Maroua Ferhat, Olesya Ilkun, Aman Makaju, Alan Achenbach, Faith Bowman, Donald L. Atkinson, William L. Holland, Ez-Zoubir Amri, Bhagirath Chaurasia, Sarah Franklin, and Sihem Boudina

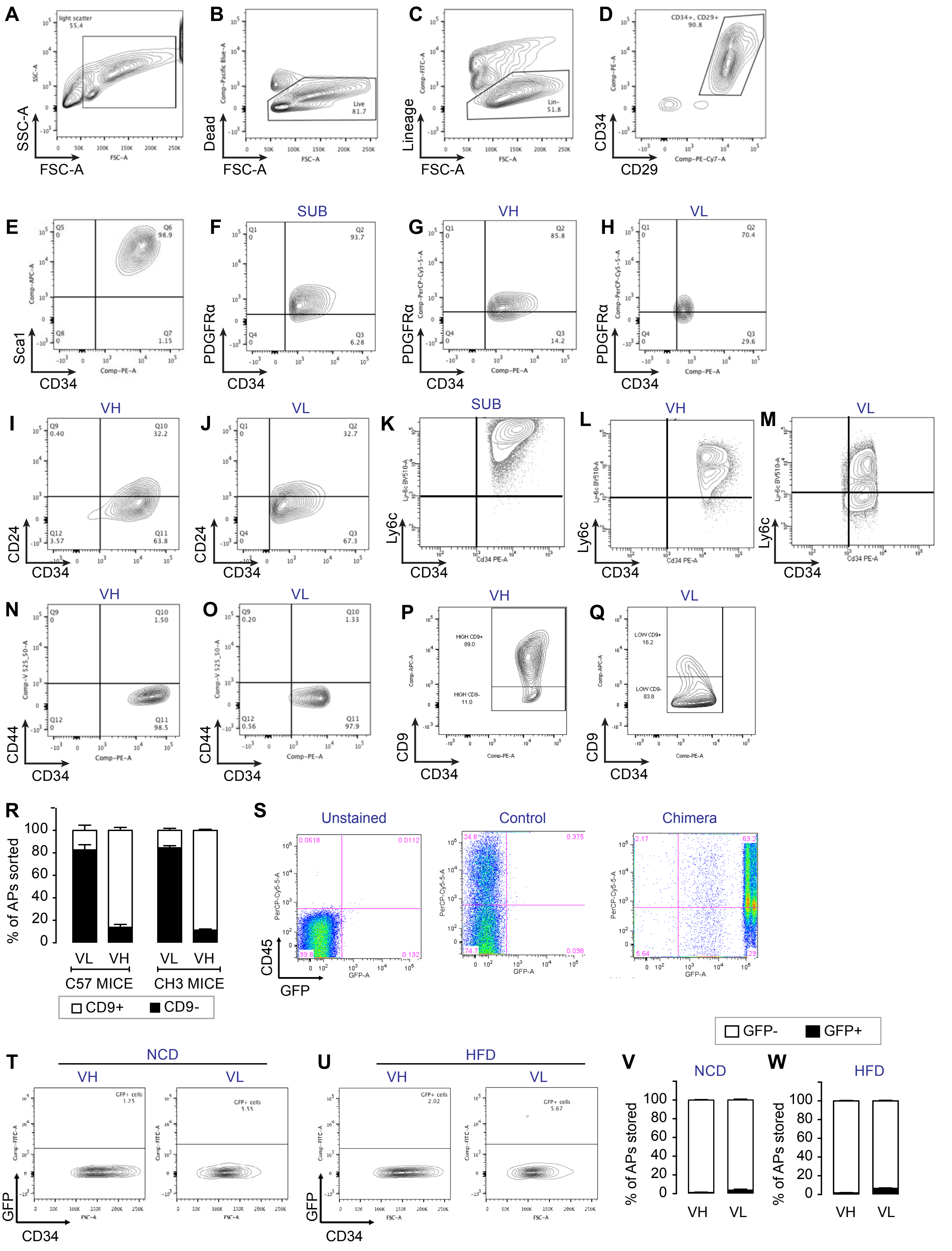
A
B
C
D
SSC-A
FSC-A
Dead
Lineage
CD34
CD29
light scatter 55.4
Live 81.7
Lin- 51.8
CD34+, CD29+ 90.8
E
F
G
H
SUB
VH
VL
Sca1
PDGFRα
Q6 98.9
Q7 1.15
Q2 93.7
Q3 6.28
Q2 85.8
Q3 14.2
Q2 70.4
Q3 29.6
I
J
K
L
M
CD24
Ly6c
Q10 32.2
Q11 63.8
Q12 3.57
Q9 0.40
Q2 32.7
Q3 67.3
N
O
P
Q
CD44
CD9
Q10 1.50
Q11 98.5
Q10 1.33
Q11 97.9
Q9 0.20
Q12 0.56
HIGH CD9+ 89.0
HIGH CD9- 11.0
LOW CD9+ 16.2
LOW CD9- 83.8
R
S
% of APs sorted
C57 MICE
CH3 MICE
CD9+
CD9-
Unstained
Control
Chimera
CD45
GFP
T
U
V
W
NCD
HFD
GFP+ cells 1.25
GFP+ cells 5.55
GFP+ cells 2.02
GFP+ cells 5.67
GFP-
GFP+
% of APs stored

Figure S1, related to Figure 1. Fluorescence activated cell sorting (FACS) of adipose progenitors (APs) and further characterization of their cell surface markers and origin. Animals were fed normal chow diet for 10 weeks. (A) Stromal vascular cells were separated based on forward and side scatter (FSC-A and SSC-A). (B) Dead cells that incorporated the dead cell stain SYTOX blue were then eliminated. (C) Lineage positive cells were eliminated by their positive staining with the lineage cocktail containing Ter119, CD31 and CD45. (D) Cells double positive for CD34 and CD29 were the sorted from the lineage negative population. (E) Cells were further sorted based on their positivity to Sca-1. (F-H) Subcutaneous, VIS low and VIS high APCs are mostly PDGFRα positive. (I-J and N-O) VIS low and VIS high APs are mainly CD24 and CD44 negative. (K-M), Subcutaneous, VIS high APs and VIS low APs were mostly Ly6c positive. (P-R) Visceral (VIS) high APs are predominantly CD9 positive whereas most of VIS low APs are CD9 negative in C57BL/6 and CH3 mice. (S) FACS plot for blood isolated from control mice or from chimera mice that were transplanted with bone marrow from UBC-GFP mice after irradiation showing that most CD45+ blood cells are also GFP+. (T-U) Bone marrow transplantation was performed, C57BL/6 recipient were maintained on normal chow diet (NCD) or high fat diet (HFD) for 8 weeks and lin-, CD29+, CD34+ and Sca1+ visceral high and visceral low APs were analyzed for GFP staining. (V-W) The proportions of GFP- and GFP+ APs in NCD and HFD conditions respectively. Values are mean ± SEM for (R), (V) and (W). n=4 mice per group. VL: Visceral low, SUB: subcutaneous, VH: VIS high.

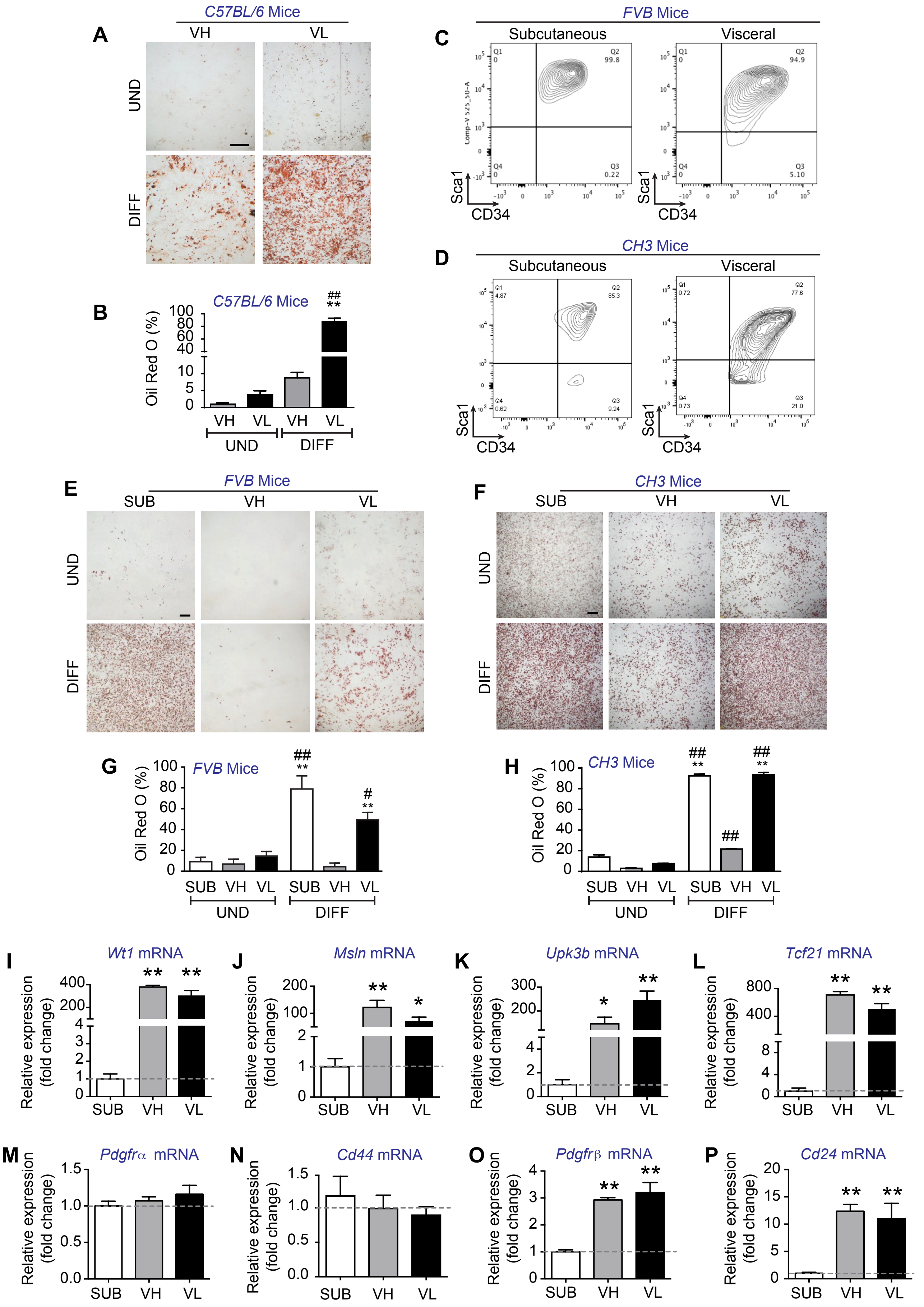
A
C57BL/6 Mice
VH
VL
UND
DIFF
B
C57BL/6 Mice
Oil Red O (%)
UND
DIFF
C
FVB Mice
Subcutaneous
Visceral
Sca1
CD34
D
CH3 Mice
Subcutaneous
Visceral
E
FVB Mice
SUB
F
CH3 Mice
G
FVB Mice
H
CH3 Mice
I
Wt1 mRNA
Relative expression (fold change)
J
Msln mRNA
K
Upk3b mRNA
L
Tcf21 mRNA
M
Pdgfrα mRNA
N
Cd44 mRNA
O
Pdgfrβ mRNA
P
Cd24 mRNA

Figure S2, related to Figure 1. Presence of VIS low and VIS high APs in other visceral depots of several mouse stains and further characterization of these cells. Animals were fed normal chow diet for 10 weeks. (A-B) Representative images of oil red O staining and the corresponding quantification in VIS low and VIS high APs isolated from retroperitoneal fat depot of C57BL/6 male mice. Cells were undifferentiated (UND) or differentiated (DIFF) for 6 days. (C-D) FACS plots of Lin-, CD29+, CD34+ and Sca1+ APs from subcutaneous and visceral fat of FVB and CH3 stains respectively. (E-F) Representative images of subcutaneous (SUB), VIS low and VIS high APs differentiation in FVB and CH3 strains respectively. (G-H) The corresponding quantification of oil red O staining presented as % red. (I-P) Relative mRNA expression of Wt1, Pdgfrα, Msln, Upk3b, Tcf21, Pdgfrα, Cd44, Pdgfrβ and Cd24 normalized by Rpl13a expression in undifferentiated subcutaneous (SUB), visceral high (VH) and visceral low (VL) APs respectively. Values are mean ± SEM *p < 0.05; **p < 0.005 versus visceral high or subcutaneous under the same condition; #p < 0.05; ##p < 0.005 versus differentiated within the same AP subtype. n=4 independent experiments, each including 10 mice). UND: undifferentiated; DIFF: differentiated; SUB: subcutaneous; VH: visceral high; VL: visceral low. Scale bar in (A), (E) and (F): 400μm.

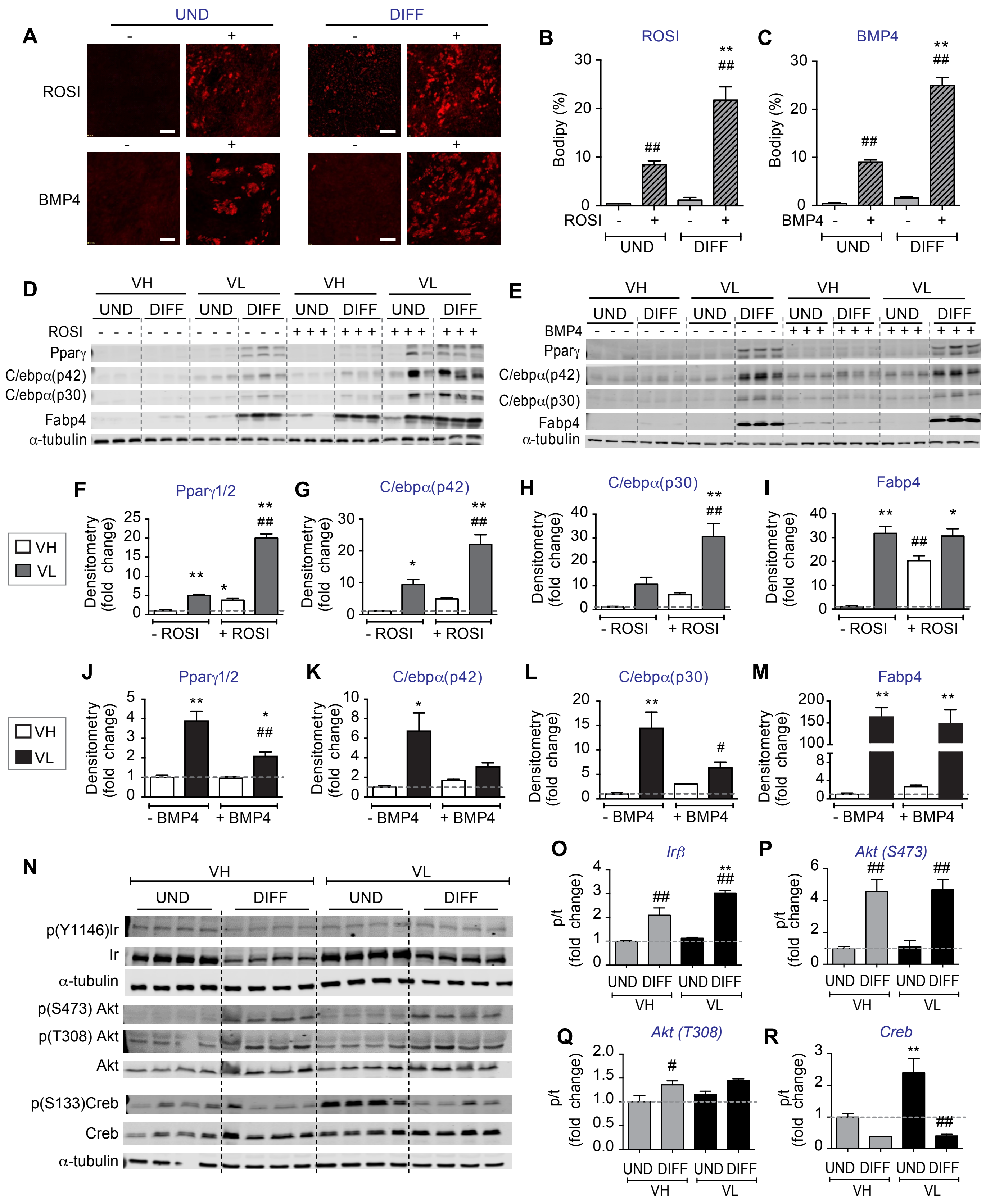
A
UND
DIFF
ROSI
BMP4
B
ROSI
Bodipy (%)
C
BMP4
D
VH
VL
UND
DIFF
Pparγ
C/ebpα(p42)
C/ebpα(p30)
Fabp4
α-tubulin
E
F
Pparγ1/2
Densitometry (fold change)
- ROSI
+ ROSI
G
C/ebpα(p42)
H
C/ebpα(p30)
I
Fabp4
J
K
L
M
- BMP4
+ BMP4
N
p(Y1146)Ir
Ir
p(S473) Akt
p(T308) Akt
Akt
p(S133)Creb
Creb
O
Irβ
p/t (fold change)
P
Akt (S473)
Q
Akt (T308)
R
Creb

Figure S3, related to Figure 1 and 2. The adipogenic potential of visceral high APs can be enhanced by rosiglitazone and BMP4 treatment. (A) Representative images of BODYPI red staining of undifferentiated (UND) and differentiated (DIFF) visceral (VIS) high APs treated with rosiglitazone (Rosi) or BMP4. (B-C) The corresponding quantification of BODYPI expressed as % red. (D-E) Representative western blots of Ppar , C/ebpα, Fabp4 and α-tubulin protein expression in VIS low and VIS high APs treated with Rosi or with BMP4 respectively. (F-M) The corresponding densitometry of Ppar , C/ebpα, Fabp4 normalized by α-tubulin and expressed as fold-changed from VH only in differentiated cells with and without Rosi or BMP4. (N) Visceral (VIS) high and low APs were sorted and cultured then differentiated. Two days post-differentiation, cells were harvested and western blots were performed for insulin receptor β (IRβ) phosphorylation on Y1146; total IRβ, Akt phosphorylation (S473 and T308); total Akt; CREB phosphorylation on S133; total CREB and α-tubulin. (O-R) The corresponding densitometry of p/total. Values are mean ± SEM. $*p < 0.05$; $**p < 0.005$ versus visceral high under the same treatment condition; $\#p < 0.05$; $\#\#p < 0.005$ versus saline treatment within the same AP subtype. n=3-4 independent experiments, each including 10 mice. Scale bar: 40μm. UND: undifferentiated; DIFF: differentiated.

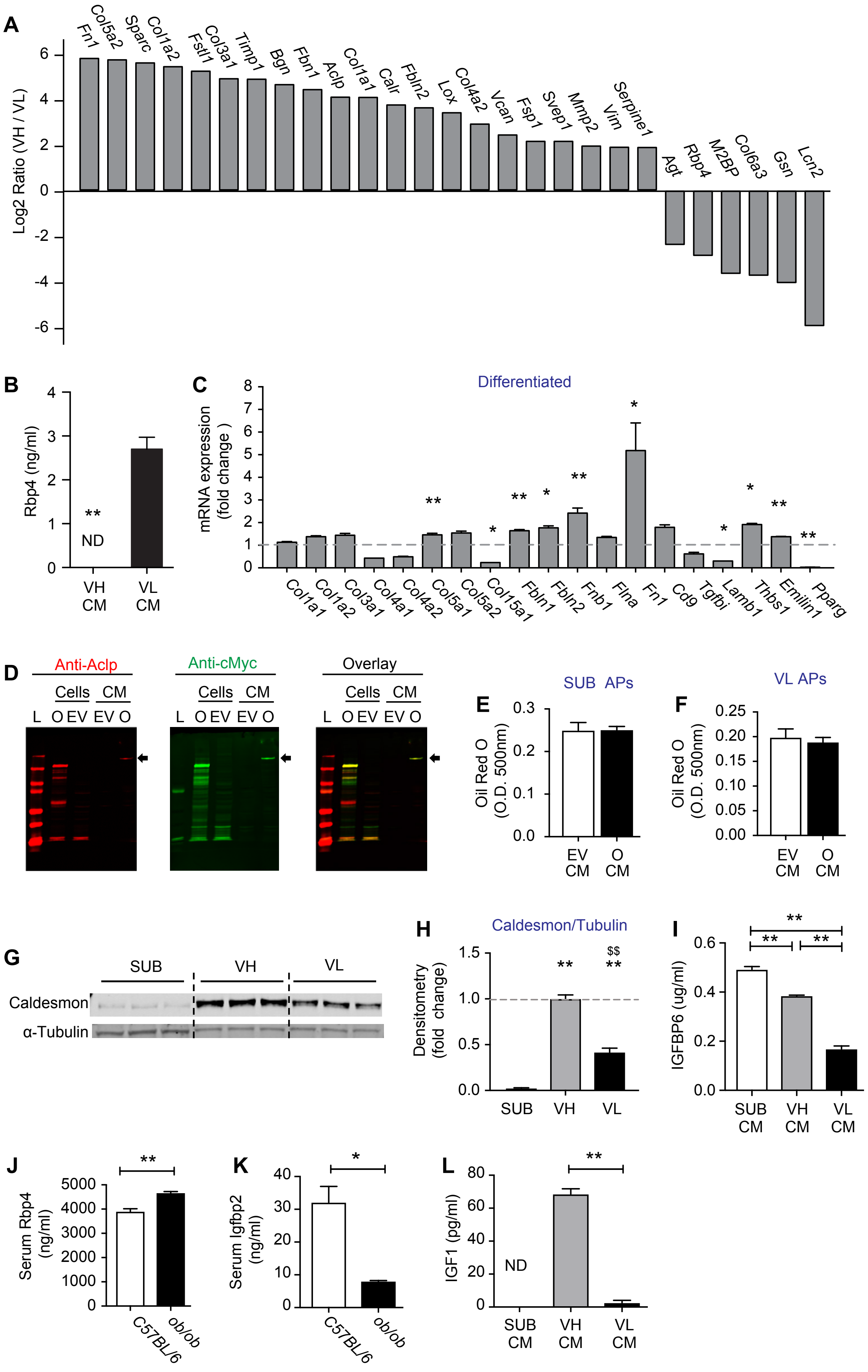
A
Log2 Ratio (VH / VL)
Fn1
Col5a2
Sparc
Col1a2
Fstl1
Col3a1
Timp1
Bgn
Fbn1
Aclp
Col1a1
Calr
Fbln2
Lox
Col4a2
Vcan
Fsp1
Svep1
Mmp2
Vim
Serpine1
Agt
Rbp4
M2BP
Col6a3
Gsn
Lcn2
B
Rbp4 (ng/ml)
**
ND
VH CM
VL CM
C
Differentiated
mRNA expression (fold change)
Col1a1
Col1a2
Col3a1
Col4a1
Col4a2
Col5a1
Col5a2
Col15a1
Fbln1
Fbln2
Fnb1
Flna
Fn1
Cd9
Tgfbi
Lamb1
Thbs1
Emilin1
Pparg
D
Anti-Aclp
Anti-cMyc
Overlay
Cells
CM
L O EV EV O
E
SUB APs
Oil Red O (O.D. 500nm)
EV CM
O CM
F
VL APs
G
SUB
VH
VL
Caldesmon
α-Tubulin
H
Caldesmon/Tubulin
Densitometry (fold change)
$$
SUB
VH
VL
I
IGFBP6 (ug/ml)
SUB CM
VH CM
VL CM
J
Serum Rbp4 (ng/ml)
C57BL/6
ob/ob
K
Serum Igfbp2 (ng/ml)
L
IGF1 (pg/ml)
ND

F**igure S4, Related to Figure 3**. Further characterization of the adipogenic inhibitor factor. (A) Secreted proteins from VH APs by LC-MS/MS proteomics. Protein abundances was quantified based on MS1 spectra intensity, and top differentially expressed proteins were plotted as Log2 ratios of VH/VL. (B) Rbp4 content in conditioned media (CM) or VIS low and VIS high APs differentiated for 6 days. (C) Relative mRNA expression of genes encoding extracellular matrix (ECM) components and fibroblast markers in 6 days differentiated VIS low and VIS high APs expressed as fold change from VIS low. (D) Representative western-blots of Aclp and cMyc in cell extracts and CM of Expi293F overexpressing mouse Aclp or an empty vector. (E-F) Subcutaneous and VIS low APs were differentiated in the presence of 10% CM from Expi293F overexpressing mouse Aclp or an empty vector for 6 days before quantification of oil red O. (G) Representative western-blots for caldesmon and α-Tubulin in 6-days differentiated (DIFF) SUB, VIS high and VIS low APs respectively. (H) The corresponding densitometry of caldesmon expression relative to α-Tubulin expressed as fold-change from VIS high. (I and L) Igfbp6 and Igf-1 content in CM of 6 days differentiated VIS SUB, VIS high and VIS low APs. (J-K) Serum Igfbp2 and Rbp4 in 12 weeks old male ob/bo mice and their corresponding C57BL/6 controls. Values are mean ± SEM. *$p < 0.05$; **$p < 0.005$ versus SUB. $$p < 0.005 versus visceral high APs. n=3-4 independent experiments, each including 10 mice. CM: conditioned media; ND: not detectable; VH: VIS high; VL: VIS low; EV: empty vector; O: overexpressed.

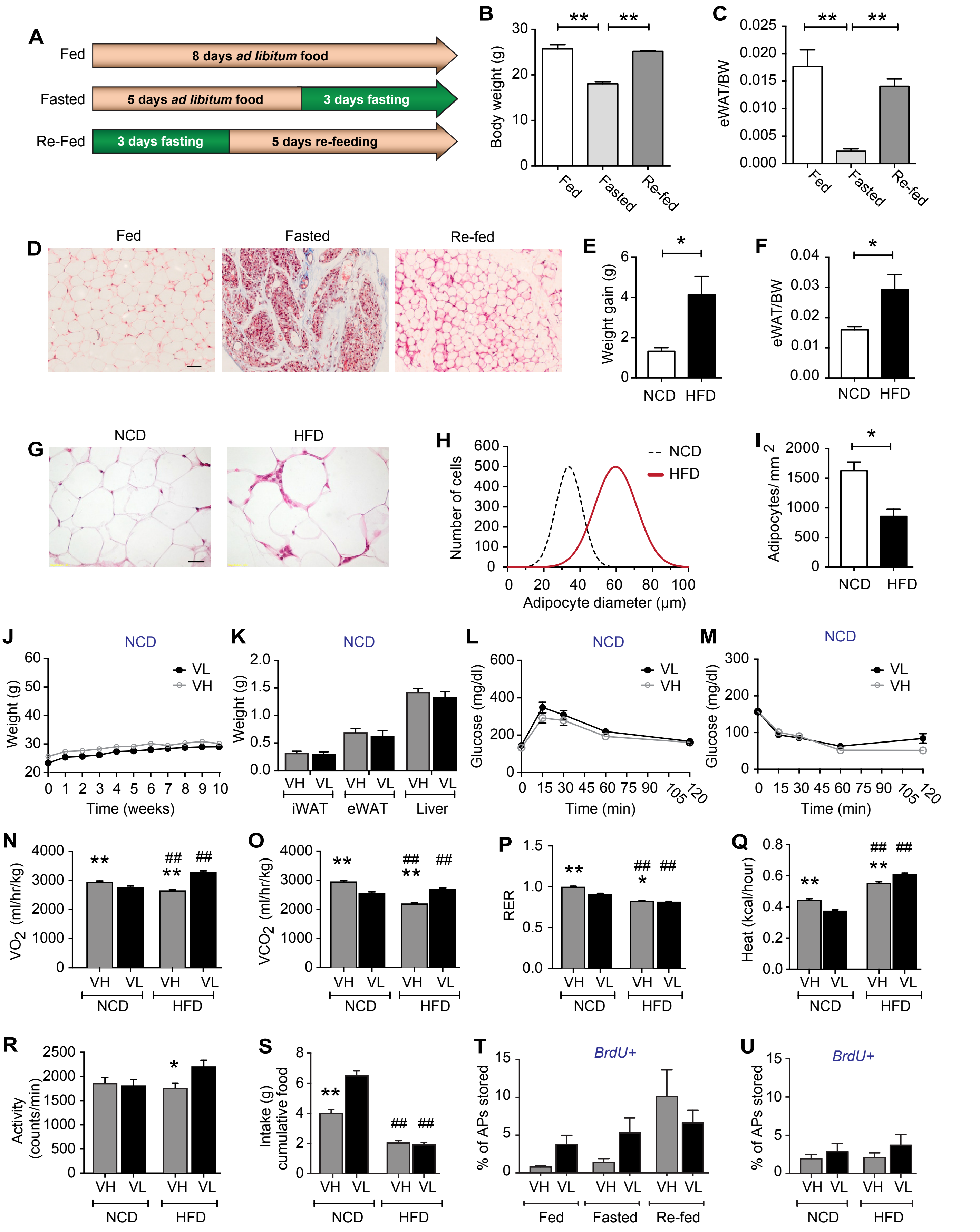
A
Fed
8 days ad libitum food
Fasted
5 days ad libitum food
3 days fasting
Re-Fed
3 days fasting
5 days re-feeding
B
Body weight (g)
Fed
Fasted
Re-fed
C
eWAT/BW
D
Fed
Fasted
Re-fed
E
Weight gain (g)
NCD
HFD
F
G
H
Number of cells
Adipocyte diameter (µm)
I
Adipocytes/ mm 2
J
NCD
Weight (g)
Time (weeks)
VL
VH
K
iWAT
eWAT
Liver
L
Glucose (mg/dl)
Time (min)
M
N
VO2 (ml/hr/kg)
O
VCO2 (ml/hr/kg)
P
RER
Q
Heat (kcal/hour)
R
Activity (counts/min)
S
Intake (g) cumulative food
T
BrdU+
% of APs stored
U

Figure S5, related to Figure 4. Fasting-refeeding and high fat diet feeding effects on body weights, fat pads weight, adipose tissue morphology and APs proliferation. (A) Fasting-refeeding protocol. (B-C) Body weights and epididymal white adipose tissue (eWAT) weight/body weight (BW) ratio in fed, fasted and re-fed C57BL/6 mice. (D) Representative eWAT sections stained with hematoxylin and eosin (H&E) in fed, fasted and refed mice. (E-F) weight gain and eWAT/BW ratios in normal chow diet (NCD) and high fat diet (HFD) fed C57BL/6 mice. (G) Representative eWAT sections in NCD and HFD mice stained with H&E. (H) Adipocyte diameter and (I) adipocyte number in NCD and HFD fed mice. (J-M) C57B6 male mice we transplanted with VIS low or VIS high cells and maintained on normal chow diet (NCD) for 10 weeks. (J) Body weights; (K) organ weights; (L) glucose tolerance tests and (M) insulin tolerance tests. (N-S) Metabolic chamber data showing whole body oxygen consumption (VO2), VCO2, respiratory exchange ratio (RER), heat production, activity and accumulated food intake over 3 days period recorded at 23 C. (T-U) Percent of BrdU positive cells in visceral (VIS) high and VIS low APs subtypes obtained from mice that were either fed ad libitum, fasted for 48 hours or fasted and refed for 5 days and from mice fed normal chow diet (NCD) or high fat diet (HFD) for 8 weeks respectively. Values are mean ± SEM. *$p < 0.05$; **$p < 0.005$. n=4-5 mice/group. Scale bar in (D): 50µm and in (G): 10µm.

Supplemental Table S1: Primer sequence for oligonucleotides used in the RT-PCR. Related to Key Resource Table.

**Oligonucleotides**		
Primers – *Cd9*:<br>F- *CTGGCATTGCAGTGCTTGCTA*<br>R- *AACCCGAAGAACAATCCCAGC*	This Paper	N/A
Primers – *Tgfbi:*<br>F- *CCTGCTTTCATCGTGGGTCC*<br>R- *CTCCAGCACGGTATTGAGTC*	This Paper	N/A
Primers – *Lamb1*:<br>F- *GAAGGGCCCCTCTCCTCTC*<br>R- *CCCATAGGGCTAGGACACCA*	This Paper	N/A
Primers – *Thbs1*:<br>F- *CCAGCTGTACATCGACTGTGA*<br>R- *GCAGATGGTAACTGAGTTCTGA*	This Paper	N/A
Primers – *Emillin1*:<br>F- *GTGTGCCTACGTGGTGACT*<br>R- *CCGGTACATGATACTTCGGGA*	This Paper	N/A
Primers – *Igfbp1:*<br>F- *TGGTCAGGGAGCCTGTGTA*<br>R- *ACAGCAGCCTTTGCCTCTT*	This Paper	N/A
Primers – *Igfbp2:*<br>F- GGCGCGGGTACCTGTGAAAAG<br>R- *TTGGGGATGTGCAGGGAGTAGAGA*	This Paper	N/A
Primers – *Igfbp3:*<br>F- GACGACGTACATTGCCTCAG<br>R- *TCTTTTGTGCAAAATAAGGCATA*	This Paper	N/A
Primers – *Igfbp4:*<br>F- GACACCTCGGGAGGAACC<br>R- *AAGAGGTCTTCGTGGGTACG*	This Paper	N/A
Primers – *Igfbp5:*<br>F- GGCGAGCAAACCAAGATAGA<br>R- *AGGTCTCTTCAGCCATCTCG*	This Paper	N/A
Primers – *Igfbp6:*<br>F- GGGCTCTATGTGCCAAACTG<br>R- *CCTGCGAGGAACGACACT*	This Paper	N/A
Primers – *Igfbp7:*<br>F- TGCCCTCCATGAAATACCAC<br>R- *GGCTGTCTGAGAGCACCTTT*	This Paper	N/A
Primers – *Acta2 (aSma):*<br>F- *ACTGGGACGACATGGAAAAG* R- *GTTCAGTGGTGCCTCTGTCA*	This Paper	N/A
Primers – *Tagln2 (Sm22a):*<br>F- *TCTTGGACGCTCTTTGCCAT*<br>R- *ATCTTCTGCTGCACCTCTCG*	This Paper	N/A

Oligonucleotides (Continued)		
Primers – *Fbln2*:<br>F- *GGACTCTGGATTCACCGACG*<br>R- *GTCTCAGGAGTCCCCGGT*	This Paper	N/A
Primers – *Fbn1*:<br>F- *CTGAGAGTCCGAGCCGCTA*<br>R- *GCCGGCAAATGGGAACAATA*	This Paper	N/A
Primers – *Flna*:<br>F- *ACAGTCACAGGTGCTGGCATT*<br>R- *GTCACTTTGCCTTTGCCTGC*	This Paper	N/A
Primers – *Fn1*:<br>F- *TGGTCCTCAAGGAACAAAGTG*<br>R- *TTCTGCATTCAACACCAAGC*	This Paper	N/A
Primers – *Wt1:*<br>F- *CCAGTGTAAAACTTGTCAGCGA*<br>R- *TGGGATGCTGCACTGTCT*	This Paper	N/A
Primers – *Cd44:*<br>F- *GAATTCTGCGCCCTCGGTT*<br>R- *TGGAATACACCTGCGTAGCG*	This Paper	N/A
Primers – *Rpl13:*	Invitrogen	Mm01612582_g1
Primers – *Msln:*	IDT	Mm.PT.58.31583786
Primers – *Upk3b:*	IDT	Mm.PT.58.30031406
Primers – *Tcf21:*	IDT	Mm.PT.58.32253118
Primers – *Pdgfra:*	IDT	Mm.PT.56a.5639577
Primers – *Pdgfrb:*	IDT	Mm.PT.56a.5869521
Primers – *Cd24:*	IDT	Mm.PT.58.13419747
